# Anti-tumorigenic properties by trichothiodystrophy mutations in melanocytic cells

**DOI:** 10.1093/narcan/zcaf026

**Published:** 2025-08-30

**Authors:** Rupesh Paudel, Lena F Sorger, Anita Hufnagel, Mira Pasemann, Tamsanqa Hove, André Marquardt, Susanne Kneitz, Andreas Schlosser, Caroline Kisker, Jochen Kuper, Svenja Meierjohann

**Affiliations:** Institute of Pathology, University of Würzburg, 97080 Würzburg, Germany; Department of Dermatology, Venereology and Allergology, University Hospital Würzburg, 97080Würzburg, Germany; Institute of Pathology, University of Würzburg, 97080 Würzburg, Germany; Institute of Pathology, University of Würzburg, 97080 Würzburg, Germany; Institute of Pathology, University of Würzburg, 97080 Würzburg, Germany; Institute of Pathology, University of Würzburg, 97080 Würzburg, Germany; Rudolf Virchow Center for Integrative and Translational Bioimaging, Institute for Structural Biology, University of Würzburg, 97080 Würzburg, Germany; Institute of Pathology, University of Würzburg, 97080 Würzburg, Germany; Klinikum Stuttgart, 70174 Stuttgart, Germany; Department of Biochemistry and Cell Biology, University of Würzburg, 97074 Würzburg, Germany; Rudolf Virchow Center for Integrative and Translational Bioimaging, Mass Spectrometry Division, University of Würzburg, 97080 Würzburg, Germany; Rudolf Virchow Center for Integrative and Translational Bioimaging, Institute for Structural Biology, University of Würzburg, 97080 Würzburg, Germany; Rudolf Virchow Center for Integrative and Translational Bioimaging, Institute for Structural Biology, University of Würzburg, 97080 Würzburg, Germany; Institute of Pathology, University of Würzburg, 97080 Würzburg, Germany

## Abstract

Germline mutations in the DNA repair helicase XPD can cause the diseases xeroderma pigmentosum (XP) and trichothiodystrophy (TTD). XP patients bear an increased risk of skin cancer including melanoma. This is not observed for TTD patients despite DNA repair defects. To examine whether TTD cells harbor features counteracting tumorigenesis, we developed a TTD melanoma cell model containing the XPD variant R722W. Intriguingly, TTD melanoma cells exhibited reduced proliferation and an increased signature of the melanocyte lineage factor MITF, along with a strong basal upregulation of REDD2, an inhibitor of the mTOR/S6K/4EBP1-dependent messenger RNA (mRNA) translation machinery. REDD2 levels were partially driven by MITF and contributed to reduced melanoma proliferation. In a TTD model for melanocytes—the progenitor cells of melanoma—the MITF gene signature was also increased, but here without affecting REDD2 expression. However, ribosomal protein synthesis was reduced particularly in R722W melanocytes after UV stress, indicating a compromised mRNA translation machinery. Impaired translation was also demonstrated for the TTD XPD variant A725P, but not for an XP variant. Concludingly, the impaired translation and reduced fitness observed in TTD melanocytes and melanoma cells, particularly after UV stress, offer a possible explanation why TTD patients do not develop melanomas.

## Graphical Abstract

**Graphical Abstract:**
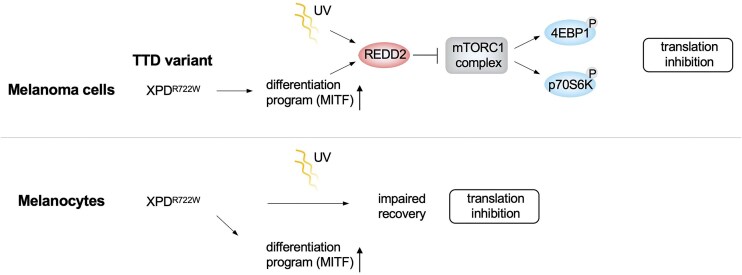

## Introduction

Our genome is constantly challenged by endogenous and exogenous agents that cause roughly 10^5^ damages per cell per day [1], requiring repair to maintain genome stability. Depending on the kind of damage, distinct systems have evolved to address and repair the respective damages. Among these mechanisms, nucleotide excision repair (NER) represents one of the most versatile with respect to the chemical nature of the repaired lesions. NER is a multistep pathway consisting of damage search and detection, damage verification, damage excision, and final repair through DNA resynthesis based on the nondamaged template strand. NER deficiency is associated with three severe hereditary diseases: xeroderma pigmentosum (XP), Cockayne syndrome (CS), and trichothiodystrophy (TTD). XP can be caused by mutations in eight gene loci (XP-A–G, XP-V). One frequently mutated gene is *ERCC2*, which represents the XP-D complementation group. The XP-D group assumes a special role, since *ERCC2* is highly associated not only with XP but in addition with a combination of XP and CS (XP/CS) as well as TTD. Consequently, one gene can be causative for three different diseases depending on the position of the mutation in *ERCC2* [2]. The protein encoded by *ERCC2* is the super family 2 helicase XPD, a central component of the general transcription factor II H (TFIIH) that plays a pivotal role in NER and transcription.

One of the hallmark features of XP is the high photosensitivity of the patients that results in a 10,000-fold and 2,000-fold increase in the risk of developing non-melanoma and melanoma skin cancer under the age of 20, respectively [3]. In contrast, TTD is characterized by developmental disorders such as impaired intelligence, short stature, and premature aging. Approximately half of the TTD patients also display photosensitivity and are defective in NER [4]. NER capacity is reduced in XPD-XP and XPD-TTD variants [5–7], with high variabilities observed among XP and TTD patient cells, respectively [8–10]. Disease symptoms vary between patients, and those with severe symptoms have a high risk of dying during childhood, mostly due to infections, while those with milder symptoms often reach adulthood. Despite their increased photosensitivity, the development of skin cancer was not reported for adult TTD patients [11], and it is unknown whether the reduced NER capacity or an impairment of transcription is related to this observation. Next to XPD, other components of the TFIIH complex are also causative for the development of photosensitive TTD when mutated. However, the helicase XPD is affected most often. Structural analyses have shown that XPD variants and their phenotype can be categorized depending on the functional impact on the protein [12–15]. XP mutations interfere with XPD helicase or ATPase activity but leave the TFIIH complex intact [6]. In contrast, TTD mutations seem to have a destabilizing effect on protein–protein interactions within the TFIIH scaffold, resulting in the destabilization of the TFIIH complex [16]. This is exemplified by the TTD variant R722W, which disrupts the interaction of XPD with the TFIIH complex component p44. The resulting destabilization of the entire complex has a major impact on both NER and *in vitro* transcriptional activity [5, 6, 17].

Due to their localization in the skin epidermis, melanocytes belong to the cell types most strongly exposed to solar UV, triggering the typical tanning response. They can transform to melanomas, which frequently arise in XP patients and are particularly dangerous due to their tendency to metastasize very early [18]. Melanomas bear a strong UV-type signature, and cumulative UV exposure is one of the main risk factors for this tumor entity, as demonstrated by epidemiological studies and mouse models (reviewed in [19]). However, it remains unclear whether melanoma development is suppressed in photosensitive TTD. We hypothesize that the analysis of TTD-causing mutations in this lineage will lead to a better understanding of cellular mechanisms that suppress melanomagenesis.

Here, we describe the generation and analysis of XPD-mutant murine melanoma cells and melanocytes as models for TTD, thereby revealing molecular insights into the effects of TTD variants on the melanocytic lineage.

## Materials and methods

### Cell culture

The murine melanoma cell line WT31, derived from Tyr; Nras^Q61K^; Ink4a^−/−^ mice [20, 21], was originally isolated in the laboratory of Owen Samson (Beatson Institute for Cancer Research, University of Glasgow, Glasgow, UK) and was a kind gift of William Faller (NKI, Amsterdam, Netherlands). Cells were maintained in Ham’s F12 (Gibco, Paisley, UK) containing 10% fetal calf serum (Sigma–Aldrich, Steinheim, Germany), 100 nM 12-*O*-Tetradecanoylphorbol-13-acetate (TPA) (Calbiochem/Merck, Darmstadt, Germany), and 1× penicillin/streptomycin (Sigma–Aldrich). Melan-a melanocytes were originally obtained from the Wellcome Trust Functional Genomic Cell Bank (Dorothy Bennett, St George’s, University of London, London, UK). For cell culture expansion, they were kept in Dulbecco’s modified Eagle medium containing 10% fetal calf serum, 200 nM TPA, and penicillin/streptomycin. #781 cells were generated from tumor tissue of *Tyr-Cre*^ERT2^;*BRAF*^V600E^;*Pten^flox/flox^* mice [22] and one round of subcutaneous injection into C57BL/6 mice. UACC-62, UACC-257, SK-MEL-2, SK-MEL-5, M19-Mel, MALME-3M, and M14 cells are part of the NCI-60 panel and were received from the NCI/NIH (DCTD Tumor Repository, National Cancer Institute at Frederick, Frederick, MD, USA). SK-MEL-28 were purchased from ATCC. Human melanoma cell lines were authenticated using the PowerPlex 16 DNA typing system (Promega, Fitchburg, WI, USA). #781 cells and all human melanoma cell lines were cultivated in Dulbecco’s modified Eagle medium (PAN Biotech) supplemented with 10% fetal calf serum (Sigma–Aldrich) and 1× penicillin/streptomycin (Sigma–Aldrich) at 37°C and 5% CO_2_. Where indicated, doxycycline (Applichem, Darmstadt, Germany), 5-azacytidine, or cycloheximide (Sigma–Aldrich) were added to the respective culture media. UV treatment was done in phosphate buffered saline (PBS) using the GS Gene Linker (Bio-Rad, Hercules, CA, USA) emitting UV radiation (254 nm) under the indicated conditions.

### Generation of expression constructs and stable transfection

To generate the XPD and REDD2 overexpression constructs, human *ERCC2* or murine *Ddit4l* was amplified via polymerase chain reaction (PCR) and cloned into the transposase vector pSB-ET-iE, which allows integration of the gene in the presence of the sleeping beauty transposase [encoded on pCMV(CAT)T7 SB100x] [23]. Cloning primers are indicated in Supplementary Table S2. For transfection, Fugene HD transfection reagent (Promega) or Lipofectamine 3000 (Invitrogen, Carlsbad, CA, USA) was used according to the manufacturer’s protocol. Positive cells were selected with 1–2 μg/ml puromycin depending on the cell lines (Calbiochem/Merck).

### Site-directed mutagenesis

To generate XPD-R722W, -A725P, and -D234N variants, *ERCC2* was cloned into the pStrataClone vector pSC-B-amp/kan (Agilent, Waldbronn, Germany). Mutations were introduced by site-directed mutagenesis using the oligonucleotides specified in Supplementary Table S2. After confirmation by Sanger sequencing (Eurofins, Ebersberg, Germany), the desired mutant ERCC2 construct was cloned into pSB-ET-iE.

### CRISPR/Cas9-mediated gene knockout

Two single guide RNA (gRNA) constructs against murine *Ercc2* were cloned using the vector pU6-(BbsI)CBh-Cas9-T2A-mCherry (Addgene #64324) for CRISPR/Cas9-mediated *Ercc2* gene knockout in murine melanoma cells WT31 and murine melan-a melanocytes. Cloning primers are shown in Supplementary Table S2. Cells were transfected with their gRNA constructs using Lipofectamine 3000 reagent (Invitrogen). Single cell clones were picked, cultured, and verified by western blot, UV sensitivity, and Sanger sequencing.

### Cell proliferation assay

Cell proliferation was evaluated by manual counting or crystal violet staining. For manual counting, cells were seeded in duplicate at equal density (3 × 10^4^ cells/plate) in 6-cm plates. Cell media were changed every 3–4 days, and cells were subcultured whenever the confluency was 70%–80%. After indicated timepoints, they were harvested by trypsinization, centrifuged, and resuspended in PBS in an appropriate volume. Cells were stained with trypan blue (Sigma–Aldrich) and viable cells, not absorbing the dye, were counted under the microscope using a hemocytometer. For crystal violet staining, cells were seeded in triplicate at equal density (3 × 10^4^ cells/well) in six-well plates and cultivated for 3 days. After that, cells were fixed in methanol, stained by 0.2% crystal violet dye in 2% ethanol for 15–20 min, and washed with PBS to remove unbound dye.

### Viability assay

Cellular viability was measured with the 3-(4,5-dimethylthiazol-2-yl)-2,5-diphenyltetrazolium bromide (MTT) assay. Cells were counted and seeded in triplicate at equal density (1–2 × 10^3^ cells/well) in 96-well plates. Three days after treatment, 5 mg/ml of MTT was added at a ratio of 1:5 (MTT:medium) to each well. After 2 h of incubation at 37°C, the medium was aspirated and 150 μl of dimethylsulfoxide (DMSO) was added to each well. The plate was then incubated at room temperature for 15 min on a shaking device. To measure formazan accumulation, the optical density at 590 nm with a reference filter of 620 nm was measured using a microplate reader (Berthold TriStar LB 941, Berthold Technologies, Bad Wildbad, Germany).

### siRNA transfection

Cells were transfected with nontargeting small interfering RNA (siRNA) or siRNA directed against murine *Mitf* or *Ddit4l*, respectively (Sigma–Aldrich). Order numbers of the respective siRNAs are indicated in Supplementary Table S1. siRNA transfection was achieved using the XtremeGene siRNA Transfection Reagent (Roche, Basel, Switzerland) in accordance with the manufacturer’s instructions. Three days after transfection, cell pellets were collected.

### RNA extraction, cDNA synthesis, and qPCR

RNA isolation was performed using TRIzol reagent (Life Technologies, Darmstadt, Germany) according to the manufacturer’s protocol. DNA digestion was performed with DNAse I (Thermo Fisher Scientific, Waltham, MA, USA) for 1 h at 37°C. Subsequently, RNA was reversely transcribed with a RevertAid First Strand complementary DNA (cDNA) Synthesis Kit (Thermo Fisher Scientific). Fluorescence-based real-time quantitative PCR (RT-qPCR) was performed and analyzed with a Mastercycler ep realplex (Eppendorf, Hamburg, Germany) or the CFX Connect™ Real-Time System (Bio-Rad Laboratories, Munich, Germany), using SYBR Green reagent (Bio-Rad). Gene expression was normalized to murine *Actb*, which remained unaltered under the treatment conditions. Oligonucleotide sequences are indicated in Supplementary Table S2.

### Cell lysis and western blot

Cells were lysed in lysis buffer (20 mM HEPES, pH 7.8, 500 mM NaCl, 5 mM MgCl_2_, 5 mM KCl, 0.1% deoxycholate, 0.5% Nonidet-P40, 10 μg/ml aprotinin, 10 μg/ml leupeptin, 200 μM Na_3_VO_4_, 1 mM phenylmethanesulfonyl fluoride, and 100 mM NaF). An equal amount (40–50 μg) of total protein was separated by 12%–14% sodium dodecyl sulfate polyacrylamide gel electrophoresis and was analyzed by western blotting. Proteins were transferred onto Amersham™ nitrocellulose membranes (GE Healthcare, Chicago, IL, USA) and blocked with 5% bovine serum albumin (Serva, Heidelberg, Germany) in Tris-buffered saline (TBS) with 0.1% Tween 20 (Roth, Karlsruhe, Germany). Primary antibody incubation was carried out overnight at 4°C. Antibodies directed against β-actin (1:5000; sc-47778) and p16 (1:500; sc-1207) were from Santa Cruz Biotechnology (Heidelberg, Germany). Antibody targeting vinculin (1:10,000; V9131) and tubulin (1:10,000; T6074) were obtained from Sigma–Aldrich (St. Louis, MO, USA). Antibodies detecting XPD (1:1000; #11963), P-p70S6K (Thr389) (1:1000; #9205), and *P*-4EBP1 (Ser65) antibody (1:1000; #9451) were received from Cell Signaling Technology (Leiden, The Netherlands). The antibodies against DDIT4L/REDD2 (1:1000; ab191096), MYC (1:5000; ab32072), and MART-1 (1:2000; ab210546) were purchased from Abcam (Cambridge, UK). The MITF antibody was a gift from C. Goding (Ludwig Institute for Cancer Research, University of Oxford, UK). For protein detection, membranes were incubated with secondary antibodies [anti-mouse HRP (1:3000; #31444, Thermo Fisher Scientific), anti-mouse Alexa Fluor™ 594 (1:5000, #A-11032, Thermo Fisher Scientific), anti-mouse Alexa Fluor™ 488 (#A-11017, Thermo Fisher Scientific), or anti-rabbit HRP (1:10,000, #170-6515, Bio-Rad)] and visualized with ECL detection Kit (Thermo Fisher Scientific) and a CCD (Kodak, Rochester, NY, USA) or Fusion SL imaging system (Vilber Lourmat, Eberhardzell, Germany). Western blot quantification was done with ImageJ. Generally, western blots show representative images of two to three independent experiments. In case of REDD2 overexpression, western blots were repeated under similar conditions in independent cell lines.

### DNA dot blot repair assay

Cells were exposed to UV as indicated. Cells were kept in PBS during UV exposure and were then transferred to their normal medium for indicated timespans, up to 16 h, before they were harvested and genomic DNA was prepared (QIAamp DNA Mini Kit, Qiagen). DNA was analyzed for the presence of 6-4 photoproducts by dot blot assay, using a specific anti-6-4 PP antibody (Cosmo Bio, Carlsbad, CA, USA), as described previously [24]. As loading control, blots were incubated with propidium iodide (Thermo Fisher Scientific).

### Statistical analysis

Unless indicated otherwise, the graphs depict the mean values of at least three independent experiments, and standard deviations are shown by error bars. Significance tests are indicated in the figure legends.

## Results

### Generation of a TTD melanoma cell model

**Figure 1.**
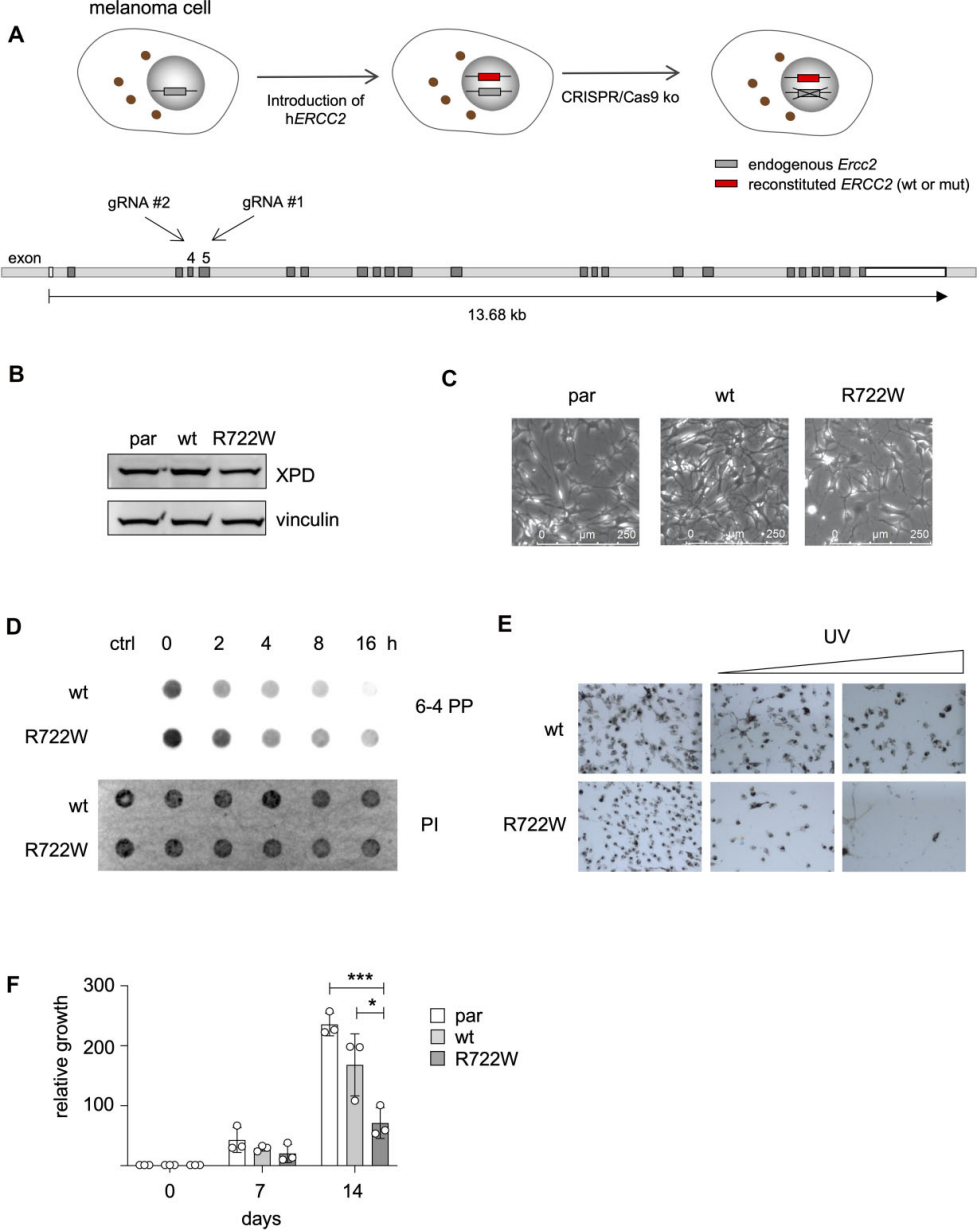
Generation of TTD-mutant melanoma cells. (**A**) Overview of the strategy for generating XPD-R722W-transgenic murine melanoma cells. As an *Ercc2* knockout is lethal, *ERCC2* expression constructs were introduced prior to knocking out the endogenous gene. Upper image: Endogenous *Ercc2* is indicated in gray. Human wild type or mutant *ERCC2* (red) were introduced by transfection before the endogenous *Ercc2* was knocked out by CRISPR/Cas9. Lower image: Visualization of the murine *Ercc2* gene and the positions of the guide RNAs. Exons are shown in white (noncoding regions) and dark gray (coding regions). Introns are depicted in light gray. (**B**) Western blot, showing the expression of XPD in parental WT31 cells (par), in WT31 cells with endogenous *Ercc2* knockout and re-expression of human wtXPD (wt), and in WT31 cells with endogenous *Ercc2* knockout and re-expression of XPD-R722W (R722W) in the presence of 30 ng/ml Dox. Please note that due to the exogenous regulation of the XPD protein in this system, XPD levels do not serve as indicator of TFIIH stability. **C**: Photographic image of par, wt, and R722W cells in culture. (**D**) DNA repair dot blot assay of XPD-wt and -R722W cells after UV exposure (1 mJ/cm^2^). Cells were harvested at indicated timepoints after UV, and genomic DNA was prepared and analyzed for the presence of 6-4 PP by dot blot, using an anti-6-4 PP antibody. “Ctrl” indicates samples that were not exposed to UV; “0 h” samples were harvested immediately after UV. Propidium iodide served as loading control. (**E**) Phase contrast images of indicated cell lines after UV exposure (with a Philips TUV 30W G30-T8 lamp, UVC radiation: 12 W) for 0, 5, and 10 s and after 7 days of recovery. (**F**) Growth of par, wt, and R722W cells (WT31). Cells were seeded in duplicate at equal density and were counted after 7 and 14 days (*n* = 3). *P < .05, ***P < .001, (Student’s *t*-test, unpaired).

XPD-R722W belongs to the most frequently reported mutations in TTD patients and was described to occur in compound heterozygous and homozygous manners [25–27]. To analyze the cellular effects of the TTD mutation without the confounding effects of different genomic backgrounds, we generated a TTD model for R722W using a melanoma cell line with engineered XPD constructs. As an *Ercc2* knockout is lethal [28], we first transfected the murine melanoma cell line WT31 with a doxycycline (Dox)-inducible human wild-type *ERCC2* (the gene encoding XPD) or R722W. After transfection, the endogenous murine *Ercc2* was knocked out via CRISPR/Cas9 (Fig. 1A), and successful gene targeting of both alleles was confirmed by sequencing. We isolated clones of WT31 cells with endogenous *Ercc2* knockout and re-expression of wild-type XPD (“wt”) as well as XPD-R722W (R722W). At a moderate concentration of 30 ng/ml Dox, XPD-wt as well as R722W expression was similar to the parental WT31 cells (“par”) (Fig. 1B). The fact that the generation of R722W melanoma cells was possible showed that TTD-causing XPD is generally compatible with *in vitro* growth of transformed cells. Also, the cellular appearance under normal cell culture conditions was similar for par, wt, and R722W cells (Fig. 1C). However, R722W cells had a prolonged persistence of genomic 6-4 photoproducts after UV exposure, as was shown by a dot blot DNA repair assay (Fig. 1D). This is in accordance with the reduced NER capacity of the R722W variant [5, 6, 29] and went along with reduced survival and recovery of R722W cells after UV treatment (Fig. 1E and Supplementary Fig. S1A). Next, we analyzed the proliferation of melanoma cells and found that R722W cells proliferated significantly slower compared to parental or reconstituted wt controls (Fig. 1F and Supplementary Fig. S1B).

### Transcriptome analysis of TTD melanoma cells

**Figure 2.**
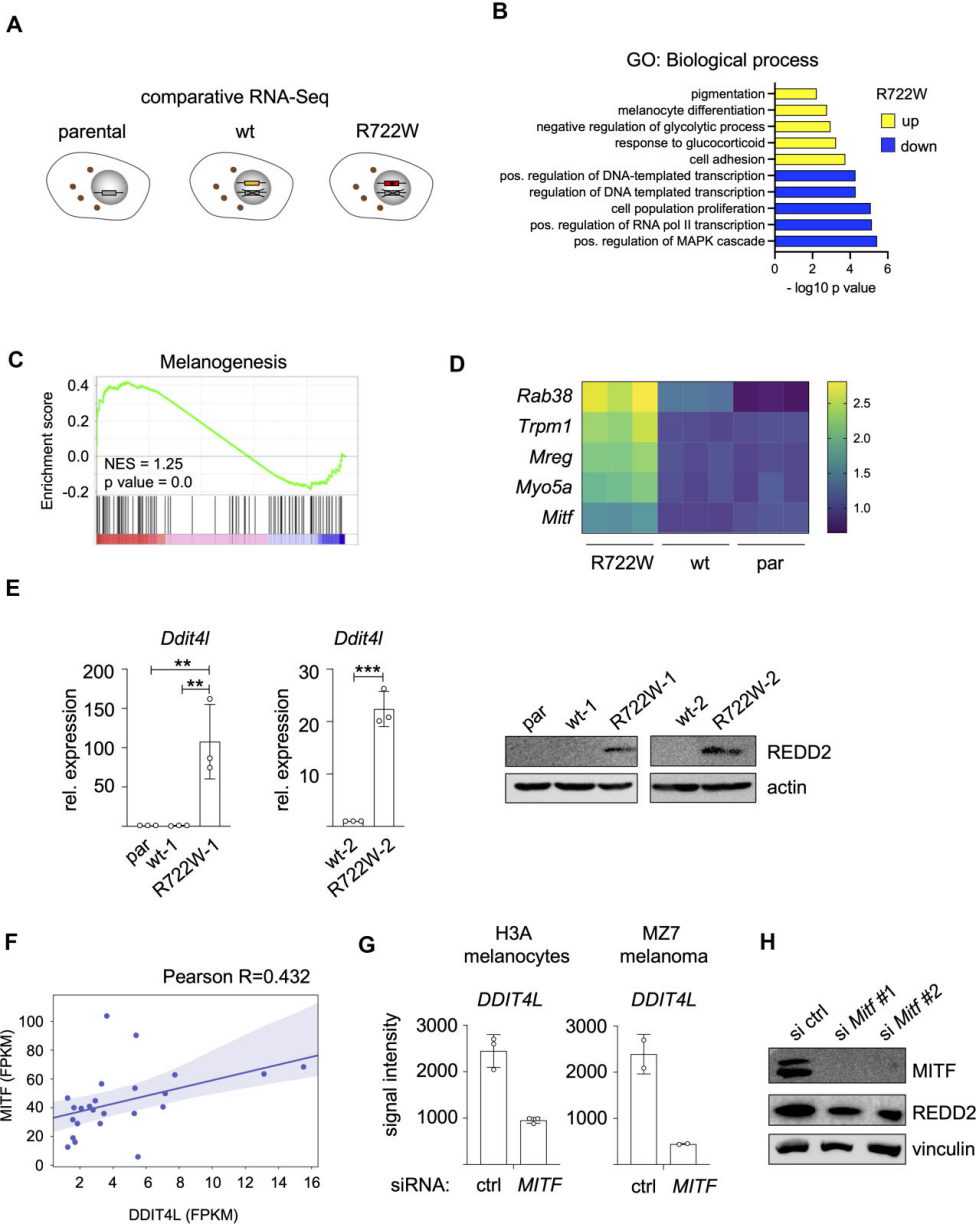
Comparative RNA sequencing of TTD-mutant melanoma cells. (**A**) Schematic illustration of the cell lines used for comparative RNA sequencing. (**B**) Top 5 significantly enriched gene groups upregulated (yellow) or downregulated (blue) in R722W melanoma cells (WT31), according to the gene ontology category “biological process” (https://davidbioinformatics.nih.gov/). (**C**) GSEA enrichment plot of the KEGG gene set “Melanogenesis,” performed on deregulated genes in XPD-R722W cells compared to their controls. (**D**) Heat plot of MITF and indicated MITF target genes in R722W, wt, and par WT31 cells, as determined by RNA sequencing. (**E**) Left: Quantification of *Ddit4l* expression by real-time PCR in two independent XPD-R722W melanoma cell clones (*n* = 3). Right: Corresponding western blot. **P < .01, ***P < .001 (Student’s *t*-test, unpaired). (**F**) Linear regression analyses of *MITF* and *DDIT4L* messenger RNA (mRNA) expression in primary skin cutaneous melanomas (TCGA) with detectable *DDIT4L* expression (fragments per kilobase of transcript per million mapped reads (FPKM) value ≥ 1) (*n* = 24). FPKM values were obtained from www.cbioportal.org. (**G**) RNA expression of *DDIT4L* after siRNA-mediated downregulation of *MITF* in human H3A melanocytes (dataset GSE61966) and MZ7 melanoma cells (dataset GSE71798). (**H**) Western blot of MITF and REDD2 in R722W cells (WT31) after siRNA-mediated knockdown of *Mitf* with two independent siRNAs.

Since an altered transcriptional profile may be causative for the phenotype of TTD cells, we performed RNA sequencing with R722W, wt, and par melanoma WT31 cells (Fig. 2A) and identified 175 expressed genes, which were commonly downregulated at least two-fold in R722W cells compared to par and wt cells, while 84 genes were upregulated (Supplementary Fig. S1C). In the group of downregulated genes, there was an enrichment of biological processes involved in transcription and proliferation. In contrast, the group of upregulated genes was enriched for cell adhesion and metabolic processes as well as melanocyte differentiation and pigmentation (Fig. 2B). The latter observation was further supported by GSEA analysis, which detected an enrichment of the gene set “Melanogenesis” (Fig. 2C). Differentiation and melanogenesis are directed by the melanocyte lineage transcription factor MITF [30, 31]. Consequently, *Mitf* as well as its target genes involved in melanin and melanosome generation were elevated in R722W cells (Fig. 2D). Unexpectedly, we could not detect changes by western blot when probing for MITF and the differentiation marker MART-1 (Supplementary Fig. S2A). This might be due to the fact that the WT31 melanoma cell line is already at the upper differentiation range, as seen by the dark color of the cell pellets (Supplementary Fig. S2B), making it harder to quantify protein changes. However, we could confirm an increase of MITF target genes in an independent set of XPD-wt and XPD-R722W clones, indicating elevated MITF activity in XPD-R722W cells (Supplementary Fig. S2C).

Beyond analyzing deregulated gene sets, we focused on individual genes and identified DNA damage inducible transcript 4-like (*Ddit4l*) as the most strongly upregulated gene in R722W cells (Supplementary Fig. S2D). *Ddit4l*encodes the protein REDD2 (“regulated in development and DNA damage response 2”), and its RNA and protein expression was specific to TTD cells, whereas it was undetectable in wt and par cells (Fig. 2E). Furthermore, WT31 cell lines engineered to express the XP-causing XPD variant D234N did not express REDD2 (Supplementary Figs S1A and S2E), suggesting that its upregulation is not a consequence of deleterious XPD variants *per se*. REDD2 is homologous to the stress-inducible REDD1 protein. Both REDD1 and REDD2 were reported to interfere with mRNA translation via mTOR signaling [32]. Notably, REDD1 loss drives KRAS-driven progression in pancreatic cancer and lung adenocarcinomas, indicating a role as tumor suppressor [33]. The function of REDD2 is far less understood and has not been investigated in the context of cancer so far. Interestingly, the corresponding gene *DDIT4L* belongs to a set of genes, whose promoter is strongly methylated in melanoma (Supplementary Fig. S3A and B). In accordance with these observations, REDD2 protein expression was either weak or undetectable in human melanoma cell lines in contrast to TTD melanoma cells (Supplementary Fig. S3C). Analysis of the TCGA skin cutaneous melanoma gene expression dataset revealed that higher *DDIT4L* expression is significantly associated with longer survival probability (Supplementary Fig. S3D), thus implying that DDIT4L/REDD2 may have a tumor-suppressive function in melanoma.

Interestingly, in cases where *DDIT4L* expression was detectable in TCGA primary melanomas, *DDIT4L* correlated with *MITF* (Fig. 2F). The link between both genes was supported by analyzing published datasets [34, 35], which revealed reduced *DDIT4L* expression in melanocytes and melanoma cells after siRNA-mediated knockdown of *MITF* (Fig. 2G). This was confirmed in R722W melanoma cells, where we downregulated endogenous MITF using two independent siRNAs and observed reduced REDD2 protein expression (Fig. 2H). In conclusion, the elevated MITF activity in TTD cells is at least partially responsible for the increase of Ddit4l/REDD2.

### The mTORC1 inhibitor REDD2 is modulated by UV and impairs melanoma cell growth

**Figure 3.**
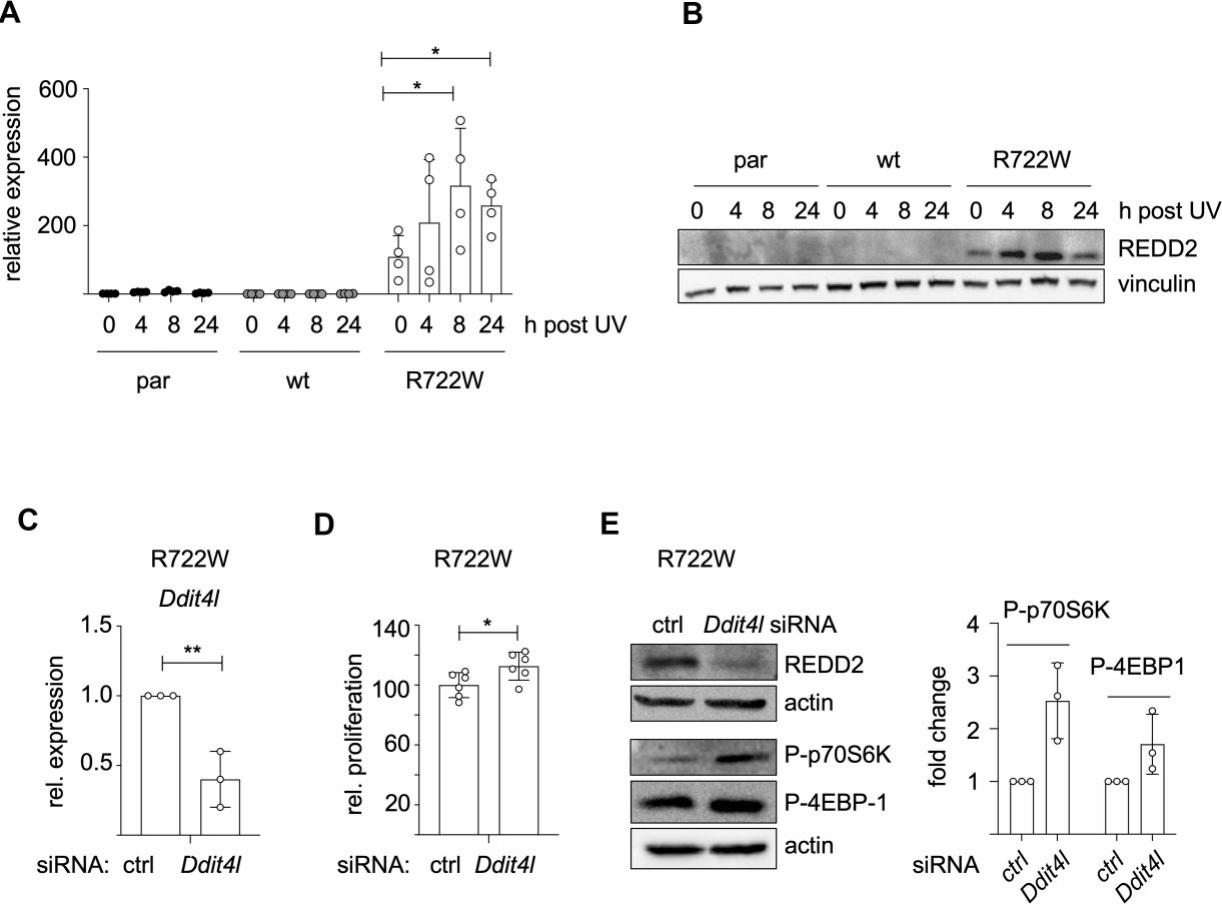
REDD2 reduces mTOR activity and viability in melanoma cells. (**A**) Real-time PCR of *Ddit4l* in indicated murine melanoma cells after exposure to UV (1 mJ/cm^2^) (*n* = 4). *P < .05 (Student’s *t*-test, unpaired). (**B**) Corresponding western blot of REDD2. (**C**) Real-time PCR, showing the *Ddit4l* knockdown efficacy in R722W murine melanoma cells (WT31) treated with control or *Ddit4l*-specific siRNA. **P < .01 (Student’s *t*-test, unpaired). (**D**) Proliferation of control and si*Ddit4l*-treated R722W cells (WT31) after 3 days of siRNA transfection. Growth was monitored by manual counting (*n* = 6). *P < .05 (Student’s *t*-test, unpaired). (**E**) Left: Representative western blot of REDD2 and the mTORC1 target proteins p70S6K (P-Thr389) and 4EBP-1 (P-Ser65) in R722W melanoma cells (WT31) treated with control or *Ddit4l*-specific siRNA. Right: Quantification of P-p70S6K and P-4EBP1 protein expressions of three independent experiments. Data were normalized to actin, and control siRNA conditions were set as 1.

Due to the fact that REDD2 is induced by cellular stress [36], we tested whether its expression in TTD cells is affected by UV, a typical source of stress in the skin. *Ddit4l* gene expression was further enhanced by UV in a transient manner only in R722W cells, which already showed basal expression of the gene, but was not induced in par and wt cells (Fig. 3A). This effect was similarly visible on the protein level (Fig. 3B) and was also seen in a second R722W cell clone (Supplementary Fig. S4A and B).

**Figure 4.**
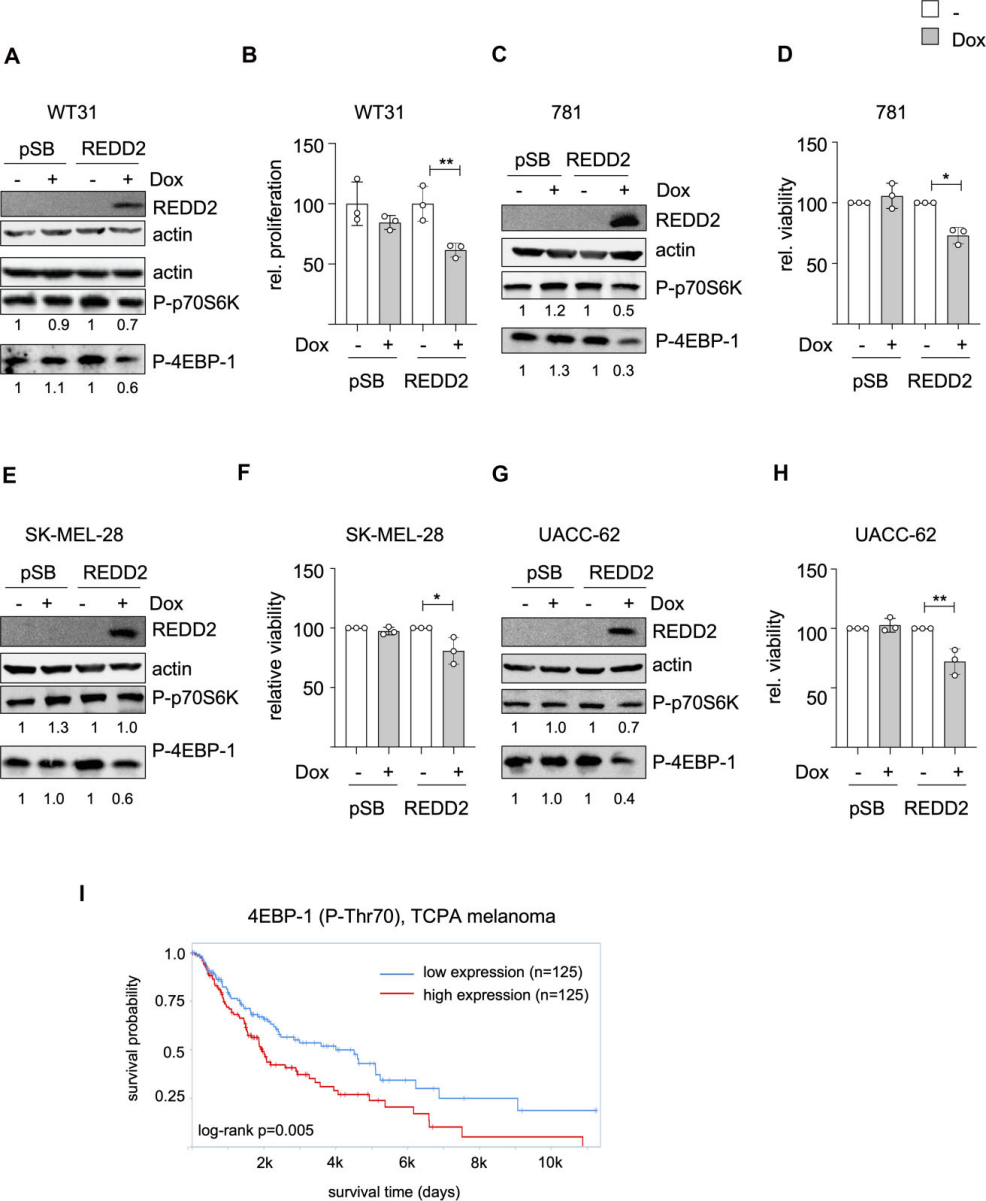
Growth-suppressive effect of REDD2 expression in independent melanoma cell lines. (**A**) Western blot of REDD2 and the mTORC1 target proteins p70S6K (P-Thr389) and 4EBP-1 (P-Ser65) in WT31 melanoma cells transfected with control vector (pSB) or an REDD2 expression vector (REDD2) in response to Dox (500 ng/ml, 24 h). (**B**) Corresponding proliferation after 3 days of Dox treatment. Growth was monitored by crystal violet staining (*n* = 3). **P < .01 (Student’s *t*-test, unpaired). (**C**) Western blot of REDD2 and the mTORC1 target proteins p70S6K (P-Thr389) and 4EBP-1 (P-Ser65) expression in 781 murine melanoma cells transfected with control vector (pSB) or an REDD2 expression vector (pSB-REDD2) in response to Dox (500 ng/ml, 24 h) (*n* = 1). (**D**) MTT viability assay showing the growth of 781 cells transfected with a vector control (pSB) or the pSB-REDD2 expression vector (REDD2) in the absence or the presence of 500 ng/ml Dox for 3 days. **E-H**: As panels C-D, but done with SK-MEL-28 (**E**, **F**) or UACC-62 (**G**, **H**) cells. Protein expression of P-p70S6K and P-4EBP1 in panels (A), (C), (E), and (G) was quantified with ImageJ as described earlier. Data in panels (D), (F), and (H) are derived from three independent experiments, each done in triplicates. *P < .05, **: P < .01 (Student’s t-test, unpaired). (**I**) Survival probability of melanoma patients according to the staining intensity of 4EBP-1 (P-Thr70), indicative of mTOR activity, in the tumor. Data were divided at the median. The analysis was done using tcpaportal (https://tcpaportal.org/tcpa/survival_analysis.html, TCGA dataset Skin Cutaneous Melanoma).

The suppression of REDD2 expression in melanoma and its association with improved overall survival suggests that REDD2 might play a tumor-suppressive role in this tumor entity. Indeed, a *Ddit4l* knockdown significantly increased the proliferation capacity of R722W cells (Fig. 3C and D), indicating that REDD2 contributes to the proliferation defect described earlier. Additionally, the knockdown led to elevated phosphorylation levels of the mTORC1 targets S70S6K (P-Thr389) and 4EBP1 (P-Ser65) (Fig. 3E), which are surrogate markers of an active cap-dependent translation [37]. Conversely, REDD2 overexpression with a Dox-inducible REDD2 expression vector led to a reduction in P-p70S6K and P-4EBP1 as well as reduced proliferation in WT31 melanoma cells (Fig. 4A and B). Similar results were obtained with the BRAF-mutant mouse melanoma cell line 781 (Fig. 4C and D). To test whether REDD2 also impairs the viability of human melanoma cell lines, we expressed *DDIT4L* in SK-MEL-28 and UACC-62 cells, which are devoid of endogenous REDD2 expression (see Supplementary Fig. S3C). For P-p70S6K, we only observed an REDD2-mediated reduction in UACC-62 cells, while SK-MEL-28 cells were unaffected. However, P-4EBP1 levels as well as cell viability were reduced in both cell lines (Fig. 4E–H). Notably, the anti-proliferative effect of REDD2 was comparable to that of the expression of CDKN2A-p16 (Supplementary Fig. S5A and B); a well-established tumor suppressor, which blocks the cell cycle by inhibiting CDK4 and CDK6 and is frequently deleted or mutated in melanoma [38, 39]. The mTOR/4EBP-1 axis is highly relevant for human melanomas, as shown by analysis of The Cancer Proteome Atlas, representing immunohistochemical stainings of 354 human melanoma samples [40]. Here, low levels of phosphorylated 4EBP1 were strongly associated with increased patient overall survival (Fig. 4I).

In summary, our studies revealed that R722W melanoma cells express the tumor suppressive REDD2, which is usually suppressed in melanoma and serves as negative regulator of mRNA translation (hereafter simply termed “translation”) by inhibiting the mTORC1/p70S6K/4EBP1 pathway. REDD2 expression was increased by MITF and UV, two triggers particularly important in the melanocytic lineage.

### Translation impairment in R722W melanocytes

**Figure 5.**
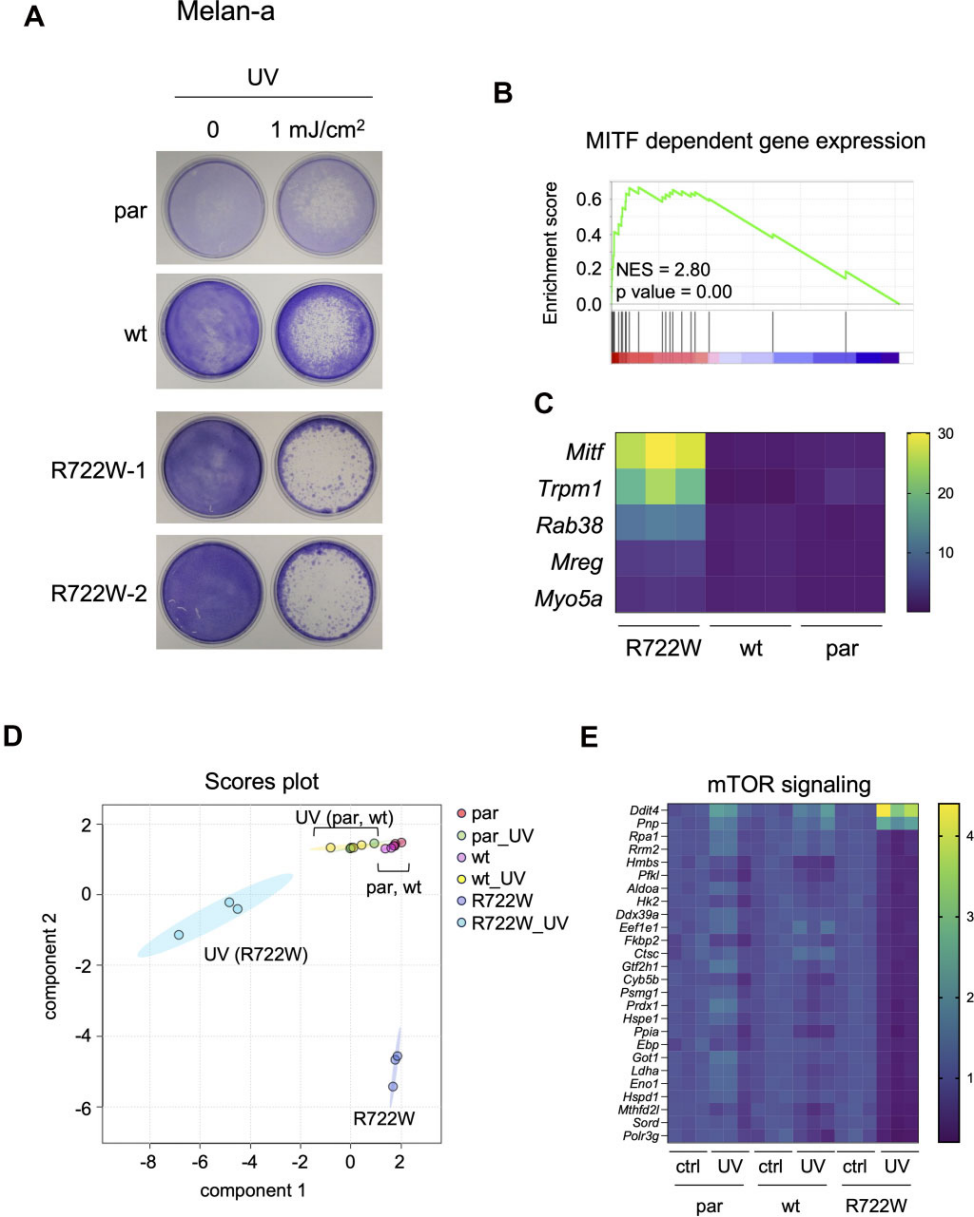
Transcriptional effects of R722W expression in melanocytes. (**A**) Crystal violet staining, showing the growth of indicated melan-a cells under control conditions or after exposure to a single dose of UV (1 mJ/cm^2^), followed by an 8-day cultivation period. Par: parental melan-a cells (expressing endogenous XPD), wt: XPD-wt reconstituted cells (as previously shown in the strategy used in melanoma cells, see Fig. 1). (**B**) GSEA enrichment plot of the gene sets “MITF-dependent gene expression” (Reactome), performed on deregulated genes in XPD-R722W melan-a cells compared to their controls (basal condition). (**C**) Heat plot of MITF and indicated MITF target genes in R722W, wt, and par melan-a cells, as determined by RNA sequencing. (**D**) Scores plot (with sPLS-DA as dimension reduction method) for indicated melan-a cells under basal conditions or 24 h after UV exposure (1 mJ/cm^2^). Each condition contained triplicates. (**E**) Heat plot showing the expression of genes from the Hallmark gene set “mTOR signaling” in the indicated cell lines. Where indicated, cells were treated with UV as described above.

To investigate whether the alterations identified in R722W melanoma cells are preserved in the melanocytic lineage, we generated R722W variant clones for the murine melanocytic cell line melan-a. Melan-a cells can be propagated *in vitro* without any further genetic modifications—in contrast to human melanocytes, which require the stable transfection of oncogenes and telomerase for long-term propagation. As expected, R722W melan-a cells had increased UV sensitivity compared to the XPD-wt counterparts (Fig. 5A). Next, gene expression of R722W and control par and wt cells was analysed by bulk RNA sequencing. Although GSEA analysis did not show a significant enrichment of the “Melanogenesis” dataset that was observed for WT31 melanoma cells (Fig. 2C), the gene set “MITF-dependent gene expression” was strongly enriched (Fig. 5B). Accordingly, *Mitf* and its melanosome and pigmentation target genes were upregulated (Fig. 5C), as shown previously for R722W WT31 cells, although to a higher degree. This went along with strongly induced protein levels of MITF and MART-1 (Supplementary Fig. S6A).

To our surprise, REDD2 protein expression was undetectable in TTD melan-a cells (Supplementary Fig. S6B). On RNA level, *Ddit4l* could be detected in low amounts only in R722W cell lines, and its expression increased after treatment with the demethylating agent 5-azacytidine (Supplementary Fig. S6C), however, without affecting protein expression (Supplementary Fig. S6D). Apparently, although MITF serves as a positive regulator of *Ddit4l*, elevated MITF activity did not enable substantial REDD2 expression in R722W melanocytes, indicating that additional factors contribute to REDD2 expression in R722W melanoma cells.

**Figure 6.**
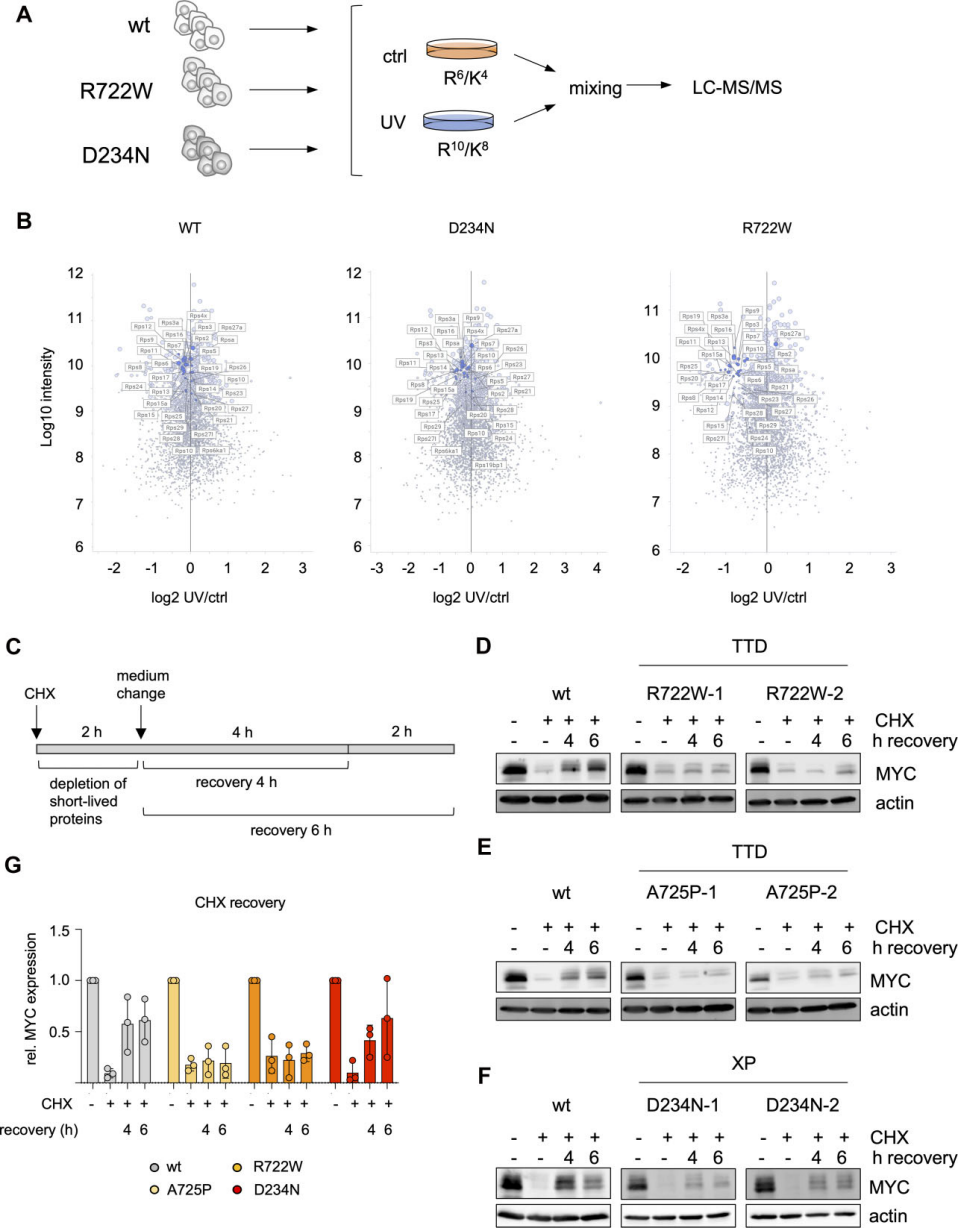
Pulsed SILAC reveals reduced RPS translation after UV treatment in melan-a cells. (**A**) Schematic representation of the experimental procedure. Controls and UV-treated samples from each indicated melan-a cell line were mixed for analysis. Data were derived from two independent replicates. (**B**) Scatter plot of the normalized log2 ratio of cellular RPS proteins in UV-treated versus control XPD-wt, XPD-D234N, or XPD-R722W melan-a cells related to the log10 protein intensity. (**C**) Overview of the protein recovery experiment after translation inhibition. CHX (10 μg/ml) was applied to the cells for 2 h. Where indicated, medium was changed afterward, and cells were kept in the absence of CHX for another 4 or 6 h to allow translation recovery. (**D-F**): Western blot showing MYC expression in melan-a cells with reconstituted XPD wild type or two independent cell clones with reconstituted XPD-R722W (**D**), XPD-A725P (**E**), or XPD-D234N (**F**). (**G**) Corresponding quantification of MYC protein expression in independent clones. Data were normalized to actin, and control conditions were set as 1.

Next, we tested to what extent stress induction affects the transcriptome of R722W melan-a cells. To acknowledge the function of melanocytes in the protection from UV damage, R722W melanocytes as well as wtXPD controls were treated with UV radiation and recovered for 24 h before being harvested. In comparison to par and wt cells, UV treatment had a much stronger transcriptional impact on R722W cells (Fig. 5D). Among the gene sets specifically altered in UV-treated R722W cells, those for cell cycle, cell division, mitosis, and DNA repair were downregulated, presumably as consequence of continued DNA damage after UV (Supplementary Fig. S6E). Interestingly, we also found a strong R722W-specific deregulation of genes of the mTOR signaling pathway, including *Ddit4* that encodes the tumor suppressor REDD1 (Fig. 5E), suggesting a dysregulation of translation. To directly investigate translation under UV stress, we performed pulsed stable isotope labeling by amino acids in cell culture (pSILAC), which measures comparative amino acid incorporation into newly synthetized proteins between different cellular states, e.g. caused by stress. As controls for R722W melan-a cells, we generated D234N (XP) mutant melan-a cells (Supplementary Fig. S7A). Shortly, melan-a cells expressing XPD-wt, XPD-R722W, and XPD-D234N were kept at similar density and were then transferred to specialized medium containing either medium or heavy stable isotopologues of arginine and lysine (R^6^/K^4^ or R^10^/K^8^, respectively) for 24 h. Cells that received R^10^/K^8^ medium were exposed to 1 mJ/cm^2^ UV directly prior to medium change (Fig. 6A) and represent the stressed condition. Both samples of each cell line were then mixed and analyzed by liquid chromatography-tandem mass spectrometry (LC–MS/MS) for the quantification of newly synthetized proteins. Interestingly, UV-dependent deregulated proteins contained many RPL members, representing proteins of the large ribosomal subunit. While RPL proteins were reduced by UV in all samples, R722W showed a stronger tendency (Supplementary Fig. S7B). Furthermore, the group of small ribosomal proteins (RPS) was clearly downregulated in R722W but only marginally in XPD-wt and D234N melan-a cells (Fig. 6B). The data thus indicate that the TTD variant R722W impairs the translation machinery, particularly under conditions of UV stress, in the melanocytic lineage. To test in an independent assay whether translation is also delayed in R722W cells irrespective of UV, we blocked translation with cycloheximide (CHX) and let the cells subsequently recover for 4 and 6 h (Fig. 6C). As readout for translation recovery, we used the short-lived transcription factor MYC, which is required for melanocyte function and is quickly degraded in the absence of translation [41, 42]. While melan-a cells with wild-type XPD recovered partially from CHX treatment within 6 h, recovery was strongly impaired in R722W cells (Fig. 6D and G). Similar results were obtained with melan-a cells expressing the TTD variant XPD-A725P. Cells with the XP variant D234N tended to show an expression recovery that resembled that of the XPD wild type, but appeared slightly delayed (Fig. 6E–G).

In conclusion, TTD mutations in XPD lead to an impairment of translation in melanoma cells and melanocytes. This is particularly pronounced under conditions of stress recovery and limits cellular fitness crucial for tumor transformation.

## Discussion

The effect of TTD mutations on the cellular and organismal phenotype is incompletely understood. However, TTD models often have one thing in common: the impaired function of highly differentiated cell types. This is thought to be due to abnormal regulation of gene transcription. The transcription defect is also discussed as underlying reason for the lack of cancer development in photosensitive TTD, as it might compromise the generation of malignant cells [43, 44]. However, previous molecular analyses of TTD cell culture and animal models frequently showed only mild effects of TTD mutations on the transcriptome and suggest that TTD mutations have different transcriptional effects in different cell types [45–48]. In livers from mice with the TTD mutation XPD-R722W, genes involved in energy metabolism and IGF1 signaling were deregulated [46], while thyroid hormone target genes were affected in the brains of TTD (XPD-R722W) mice [49]. In addition, distinct genes with high transcriptional demand were dysregulated in specialized cell types, as e.g. shown for the expression of beta globin in erythroid cells [50] or the immunoglobulin subunit IGH in B cells [51]. Furthermore, TTD patient fibroblasts show strongly reduced levels of prostaglandin I_2_ synthase [48], an enzyme usually expressed at high levels in this cell type due to the substantial role of fibroblasts in prostacyclin synthesis [52].

In our present study, we show that melanoma cells tolerated the replacement of endogenous XPD with XPD-R722W, demonstrating that the TTD mutation did not interfere with vital tumor cell functions at least under *in vitro* conditions. Transcriptional deregulation was rather mild, in accordance with previous work, but an upregulation of melanocytic differentiation genes was detected in melanoma cells as well as in an independent melanocyte TTD model.

The melanocytic gene set is driven by the transcription factor MITF, which enhanced the levels of the tumor suppressor and translation inhibitor REDD2 in the TTD melanoma cells, where it antagonized proliferation. Although REDD2 was not expressed in TTD melanocytes, these cells still showed impaired translation, particularly after recovery from stress such as UV exposure or CHX. Melanoma and melanocyte TTD models thus shared an increased differentiation in gene expression as well as translation impairment. Future studies are required to further delineate the underlying mechanisms. While REDD2 is clearly involved in limiting translation in TTD melanoma cells, other factors control the translation impairment in TTD melanocytes. It remains to be determined which role MITF and the differentiation phenotype play in this scenario.

Accumulating evidence, mostly derived from non-photosensitive TTD (NPS-TTD) models, sheds light on the effects of TTD-causing mutations on processes beyond transcription. Mutations in *TTDN1/MLKIP1*, which is mutated in a significant subgroup of NPS-TTD, have splicing defects that results in proteome alterations [53]. Other forms of NPS-TTD, caused by mutations in the TFIIE subunit β or deleterious mutations in the aminoacyl transfer RNA (tRNA) synthetases AARS1, MARS1, TARS1, and CARS1, are causative for reduced ribogenesis and a dysbalance in ribosomal protein expression [54] as well as reduced translation and stability, respectively [55–57]. However, there are also links between TFIIH, where mutations of photosensitive TTD (PS-TTD) are located, and translation. Recent studies demonstrated that human TTD fibroblasts (carrying the XPD variant R112H) have impaired ribosomal fidelity, which was not observed for XP fibroblasts [58]. TFIIH was previously described to be involved in ribosome biogenesis by promoting RNA polymerase I-dependent ribosomal RNA (rRNA) elongation as well as rRNA maturation. In line with this, XPD-R722W as well as *Ttda*-knockout cells showed rRNA transcription defects [59, 60]. As it is known that UV damage delays rRNA synthesis by stalling RNA polymerase I on the damaged DNA [61], one would assume that the negative effects of the TTD variants are enhanced in the presence of UV damage. We observed in the present study a reduced *de novo* synthesis of ribosomal proteins after UV exposure, which was particularly pronounced in R722W melanocytes. Furthermore, the delayed recovery of MYC protein levels after CHX treatment supports the observation that the TTD mutants suffer from reduced resumption of translation after stress independent of UV stress. Data from other photosensitive TTD models have led to the conclusion that TTD mutants show mild phenotypes in unperturbed growth conditions but strong alterations under stress conditions [62, 63]. In the context of melanoma, recovery from stress encountered in the tumor niche and robust protein synthesis are important for tumor maintenance. Inhibition of translation, e.g. by blockage of translation initiation combined with amino acid limitation, has an anti-tumorigenic effect on melanoma in mouse models [64]. In general, the translational machinery is considered to be a valuable therapeutic target for different cancer entities and can be addressed by the translation elongation inhibitor omacetaxine or mTOR inhibitors, respectively, which are currently tested in numerous clinical cancer studies [65].

In summary, our data provide the first functional link between TTD and the suppression of melanoma growth, marked by dysregulated translation, and provides a possible explanation for the lack of cancer predisposition in TTD patients beyond a global effect on transcription.

## Supplementary Material

zcaf026_Supplemental_Files

## Data Availability

RNA sequencing data were deposited in the Sequence Read Archive from NCBI (PRJNA662765 and PRJNA1200305). Original western blots have been deposited at Mendeley Data Repository (https://data.mendeley.com/datasets/yjr3fdbjp9/1). Pulsed SILAC data are presented in Supplementary Table S3.
