# Supplementary material for: Anti-tumorigenic properties by trichothiodystrophy mutations in melanocytic cells
Source: NAR Cancer. 2025 Aug 30;7(3):zcaf026. doi: 10.1093/narcan/zcaf026 (PMC12409403; doi:10.1093/narcan/zcaf026)
Supplement: zcaf026_Supplemental_Files [file zcaf026_supplemental_files.zip › Supplementary Information and figures.pdf]

Supplementary Information

Supplementary Methods

RNA sequencing

WT31 cells stably expressing wild-type and mutant XPD constructs along with parental cells were cultivated in triplicate and were kept for 3 days in presence of 30 ng/ml doxycycline. Total RNA was extracted with RNeasy Kit (Qiagen, Hilden, Germany). RNA integrity was assessed with a Bioanalyzer 2100 (Agilent). RNA integrity numbers (RINs) of RNA pools ranged between 8.8 and 9.8 (RNA integrity number 10 corresponding to completely intact RNA). Libraries for RNA-Seq samples were constructed using 500 ng total RNA following the manufacturer's instructions using the NEBNext® UltraTM II RNA Library Prep Kit for Illumina® (NEB, Frankfurt, Germany) and sequenced on a NextSeq 500 (Illumina, Berlin, Germany). Thirty-two to thirty-nine million strand-specific short reads were obtained for each RNA-Seq library. Library preparation and sequencing was performed at the Core Unit Systems Medicine at the University of Würzburg.

For transcriptome analysis, reads were mapped to the *Mus_musculus* GRCm38.88 reference genome using the RNA-sequence aligner STAR (https://github.com/alexdobin/STAR/releases). Transcripts were quantified as expected read counts using RSEM (http://deweylab.github.io/RSEM) and differentially expressed genes were detected by DESeq2 (Bioconductor/R) (1). Genes were considered to be differentially expressed, if they reached p.values ≤ 0.05 and a log2fold change of ≥ |1|. Functional clustering was made using DAVID 6.7 analysis (https://david-d.ncifcrf.gov/) (2,3).

Melan-a cells stably expressing wild-type and mutant XPD constructs along with parental cells were seeded at equal density (1.2 x 10^6 cells/10 cm dish) in triplicate in the presence of 250 ng/ml doxycycline. Cells were exposed to UV as indicated. Total RNA was extracted with RNeasy Kit (Qiagen). RNA quality was checked using a 5200 Fragment Analyzer with the DNF-471-33 - SS Total RNA 15nt kit (Agilent Technologies). The RQN for all samples was

between 5.4 and 8.5. DNA libraries suitable for sequencing were prepared from 300 ng of total RNA with oligo-dT capture beads for poly-A-mRNA enrichment using the TruSeq Stranded mRNA Library Preparation Kit (Illumina) according to manufacturer's instructions. After 15 cycles of PCR amplification, the size distribution of the barcoded DNA libraries was estimated ~340 bp by electrophoresis on 5200 Fragment Analyzer with the DNF-474-33 - HS NGS Fragment 1-6000bp kit (Agilent Technologies). Sequencing of pooled libraries, spiked with 1% PhiX control library, was performed at ~25 million reads/sample in single-end mode with 100 nt read length on the NextSeq 2000 platform (Illumina). Demultiplexed FASTQ files were generated with bcl-convert v4.3.6 (Illumina). Gene Set Enrichment Analysis (GSEA) was performed using the Molecular Signatures Database (MSigDB) (4,5).

Pulsed SILAC

For pulsed SILAC analysis, cells were shortly transferred to PBS, where they were left untreated or were treated with UV (1 mJ/cm^2). Afterwards, they were transferred to DMEM medium for SILAC (88370, Thermo Fisher Scientific) in the presence of 10% dialysed serum (Thermo Fisher Scientific), penicillin/streptomycin and 1 mM L-arginine hydrochloride and 0.5 mM L-lysine hydrochloride. Arg6 (CLM-2265-H, Cambridge Isotope Laboratories, Tewksbury, MA, USA) and Lys4 (88438, Thermo Fisher Scientific) were used for controls, while Arg10 (CNLM-539-H, Cambridge Isotope Laboratories) and Lys8 (CNLM-291-H, Cambridge Isotope Laboratories) were used for cells undergoing UV treatment. Cells were cultivated for 24 h before harvesting.

Gel electrophoresis

Cell pellets with 1x10^6 cells were resuspended in 400 µl lysis buffer, as indicated above. After 45 min on ice and Bioruptor (Diagenode, Seraing, Belgium) treatment with 5 cycles of 30 s the samples were centrifuged 10 min at 14 000 g. Half of the supernatant was precipitated with a fourfold volume of acetone overnight at -20°C. Pellets were washed with acetone at -20°C. Precipitated proteins were dissolved in NuPAGE® LDS sample buffer (Life Technologies,

Darmstadt, Germany), reduced with 50 mM DTT at 70°C for 10 min and alkylated with 120 mM iodoacetamide at room temperature for 20 min. Separation was performed on NuPAGE® Novex® 4-12% Bis-Tris gels (Life Technologies) with MOPS buffer according to the manufacturer's instructions. A sample amount corresponding to $5x10^5$ input cells was loaded on the gel. The gel was washed three times for 5 min with water and stained for 1 h with Simply Blue™ Safe Stain (Life Technologies). After washing with water for 1 h, each gel lane was cut into 15 slices.

In-gel digestion

The excised gel bands were destained with 30% acetonitrile in 0.1 M NH_4HCO_3 (pH 8), shrunk with 100% acetonitrile, and dried in a vacuum concentrator (Concentrator 5301, Eppendorf). Digests were performed with 0.1 µg trypsin (Trypsin Gold, Mass Spectrometry Grade, Promega) per gel band overnight at 37°C in 0.1 M NH_4HCO_3 (pH 8). After removing the supernatant, peptides were extracted from the gel slices with 5% formic acid, and extracted peptides were pooled with the supernatant.

NanoLC-MS/MS analysis

NanoLC-MS/MS analyses were performed on an Orbitrap Fusion (Thermo Fisher Scientific) equipped with a PicoView Ion Source (New Objective, Littleton, CO, USA) and coupled to an EASY-nLC 1000 (Thermo Fisher Scientific). Peptides were loaded on a trapping column (2 cm x 150 µm ID, PepSep) and separated on capillary columns (30 cm x 150 µm ID, PepSep) both packed with 1.9 µm C18 ReproSil and separated with a 45 min linear gradient from 3% to 30% acetonitrile and 0.1% formic acid and a flow rate of 500 nl/min.

Both MS and MS/MS scans were acquired in the Orbitrap analyzer with a resolution of 60 000 for MS scans and 30 000 for MS/MS scans. HCD fragmentation with 35% normalized collision energy was applied. A Top Speed data-dependent MS/MS method with a fixed cycle time of 3 s was used. Dynamic exclusion was applied with a repeat count of 1 and an exclusion duration of 30 s; singly charged precursors were excluded from selection. Minimum signal

threshold for precursor selection was set to 50 000. Automatic gain control (AGC) was used with the manufacturer's standard settings for MS scans and MS/MS scans. EASY-IC was used for internal calibration.

MS data analysis

Raw MS data files were analyzed with MaxQuant version 1.6.2.2. Database search was performed with Andromeda, which is integrated in the utilized version of MaxQuant (6). The search was performed against the UniProt mouse (June 4, 2021, UP000000589, 55341 entries). Additionally, a database containing common contaminants was used. The search was performed with tryptic cleavage specificity with 3 allowed miscleavages. Protein identification was under control of the false-discovery rate (FDR; <1% FDR on protein and peptide spectrum match (PSM) level). In addition to the MaxQuant default settings, the search was performed against the following variable modifications: Protein N-terminal acetylation, Gln to pyro-Glu formation (N-term. Gln) and oxidation (Met). Carbamidomethyl (Cys) was set as fixed modification. Arg6 and Lys4 were set for medium SILAC labels and Arg10 and Lys8 for heavy SILAC labels. Further data analysis was performed using R scripts developed in-house. For quantification of pSILAC-labeled proteins, the median was calculated from log2-transformed normalized peptide heavy-to-medium ratios (H/M) for each protein. Two ratio counts were required for protein quantification. The mean H/M protein ratio was calculated from 2 independently harvested replicates for WT, R722W and D234N variants. Original data are shown in Supplementary Table 3.

References

1. Love, M.I., Huber, W. and Anders, S. (2014) Moderated estimation of fold change and dispersion for RNA-seq data with DESeq2. *Genome Biol*, **15**, 550.
2. Huang da, W., Sherman, B.T. and Lempicki, R.A. (2009) Systematic and integrative analysis of large gene lists using DAVID bioinformatics resources. *Nat Protoc*, **4**, 44-57.
3. Huang da, W., Sherman, B.T. and Lempicki, R.A. (2009) Bioinformatics enrichment tools: paths toward the comprehensive functional analysis of large gene lists. *Nucleic Acids Res*, **37**, 1-13.
4. Subramanian, A., Tamayo, P., Mootha, V.K., Mukherjee, S., Ebert, B.L., Gillette, M.A., Paulovich, A., Pomeroy, S.L., Golub, T.R., Lander, E.S. *et al.* (2005) Gene set enrichment analysis: a knowledge-based approach for interpreting genome-wide expression profiles. *Proc Natl Acad Sci U S A*, **102**, 15545-15550.
5. Milacic, M., Beavers, D., Conley, P., Gong, C., Gillespie, M., Griss, J., Haw, R., Jassal, B., Matthews, L., May, B. *et al.* (2024) The Reactome Pathway Knowledgebase 2024. *Nucleic Acids Res*, **52**, D672-D678.
6. Cox, J. and Mann, M. (2008) MaxQuant enables high peptide identification rates, individualized p.p.b.-range mass accuracies and proteome-wide protein quantification. *Nat Biotechnol*, **26**, 1367-1372.
7. Ghandi, M., Huang, F.W., Jane-Valbuena, J., Kryukov, G.V., Lo, C.C., McDonald, E.R., 3rd, Barretina, J., Gelfand, E.T., Bielski, C.M., Li, H. *et al.* (2019) Next-generation characterization of the Cancer Cell Line Encyclopedia. *Nature*, **569**, 503-508.

Supplementary Figures

Supplementary Figure 1

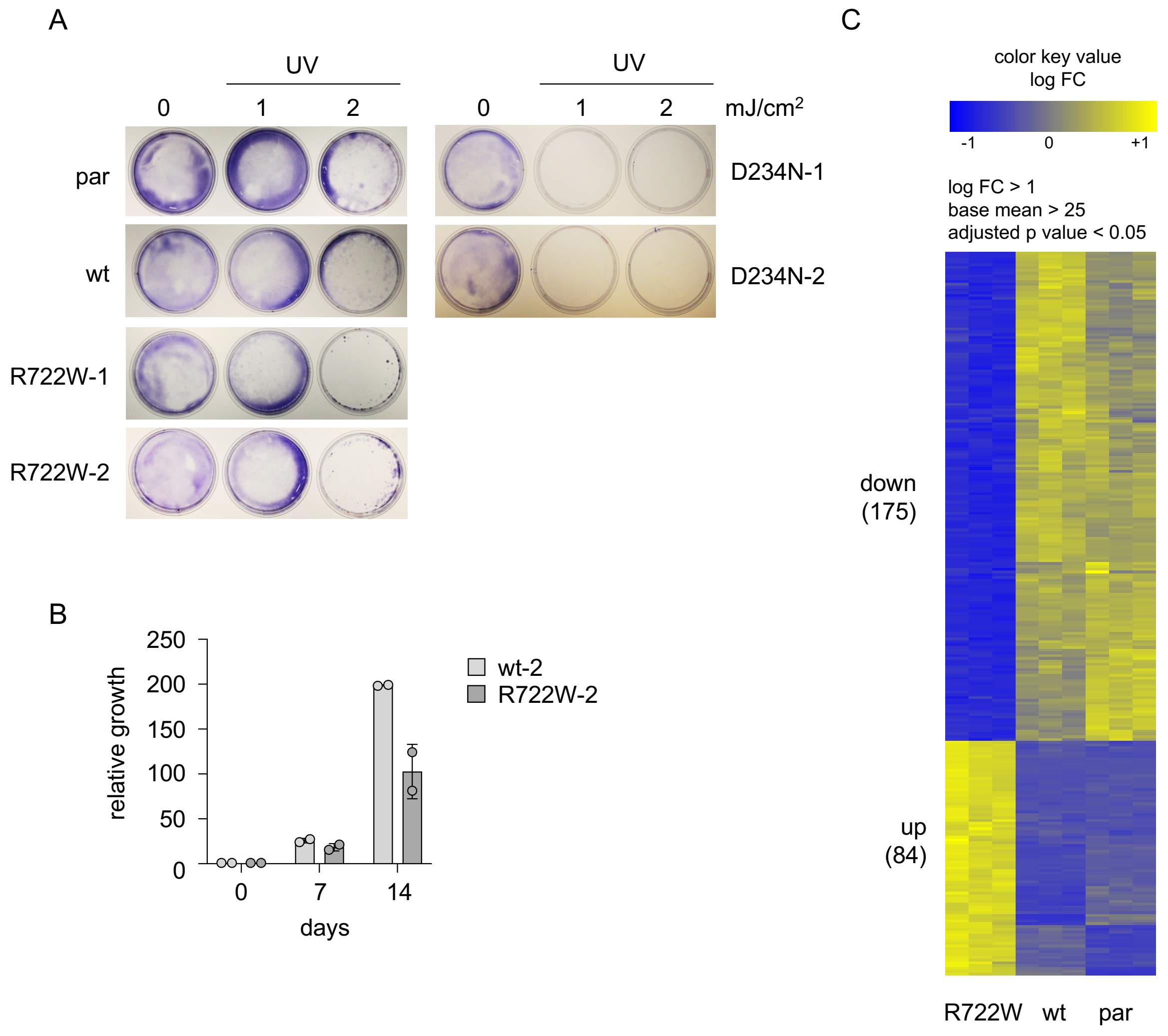

Supplementary Figure 1: Altered basal gene regulation in TTD cells

A: Crystal violet staining, showing the growth of indicated WT31 cells under control conditions or after exposure to a single dose of UV (1 and 2 mJ/cm^2), followed by an 8-day cultivation period. Please note that for each cell line the controls, but not UV-treated samples, were split 1:4 during this time span. **B:** Growth of wt-2 and R722W-2 WT31 cells, representing independent clones complementing those shown in Figure 1. Cells were seeded in duplicate at equal density and were counted after 7 and 14 days (n = 2). **C:** Heat plot of genes, which were expressed at a base mean >25 and were deregulated at least two-fold in R722W WT31 cells compared to wt and par cells (derived from RNA sequencing as described in Figure 2A).

Supplementary Figure 2

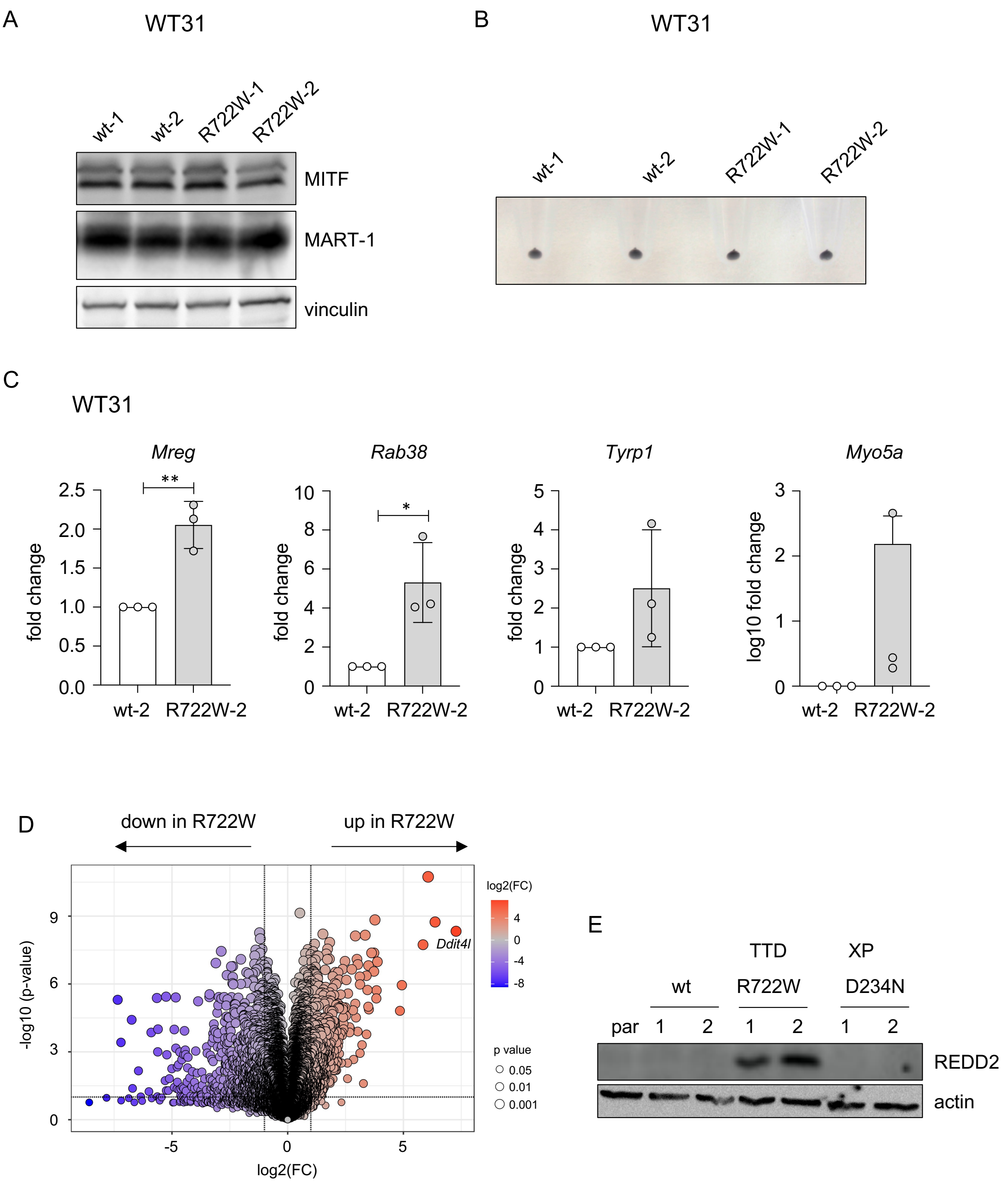

Supplementary Figure 2: Deregulated genes in TTD cells

A: Protein blot of the melanocyte lineage marker MITF and the differentiation marker MART-1 in two XPD-wt and two XPD-R722W clones (WT31 melanoma cell line). Vinculin served as loading control. **B:** Visual appearance of similar-sized cell pellets of the corresponding cell clones. **C:** Real-time PCR of MITF target genes *Mreg*, *Rab38*, *Tyrp1* and *Myo5a* (n=3). *:

p<0.05; **: p<0.01 (Student`s t test, unpaired). Please note that *Tyrp1* and *Myo5a* expression did not meet significance standards due to high standard derivation between experiments, despite their elevated expression in R722W in every experiment. **D:** Volcano plot representing deregulated genes from R722W cells versus wt and par cells. **E:** Western blot, showing REDD2 expression in parental WT31 melanoma cells (par), as well as two clones of XPD-wt, XPD-R722W and XPD-D234N WT31 melanoma cells, respectively. Actin served as loading control.

Supplementary Figure 3

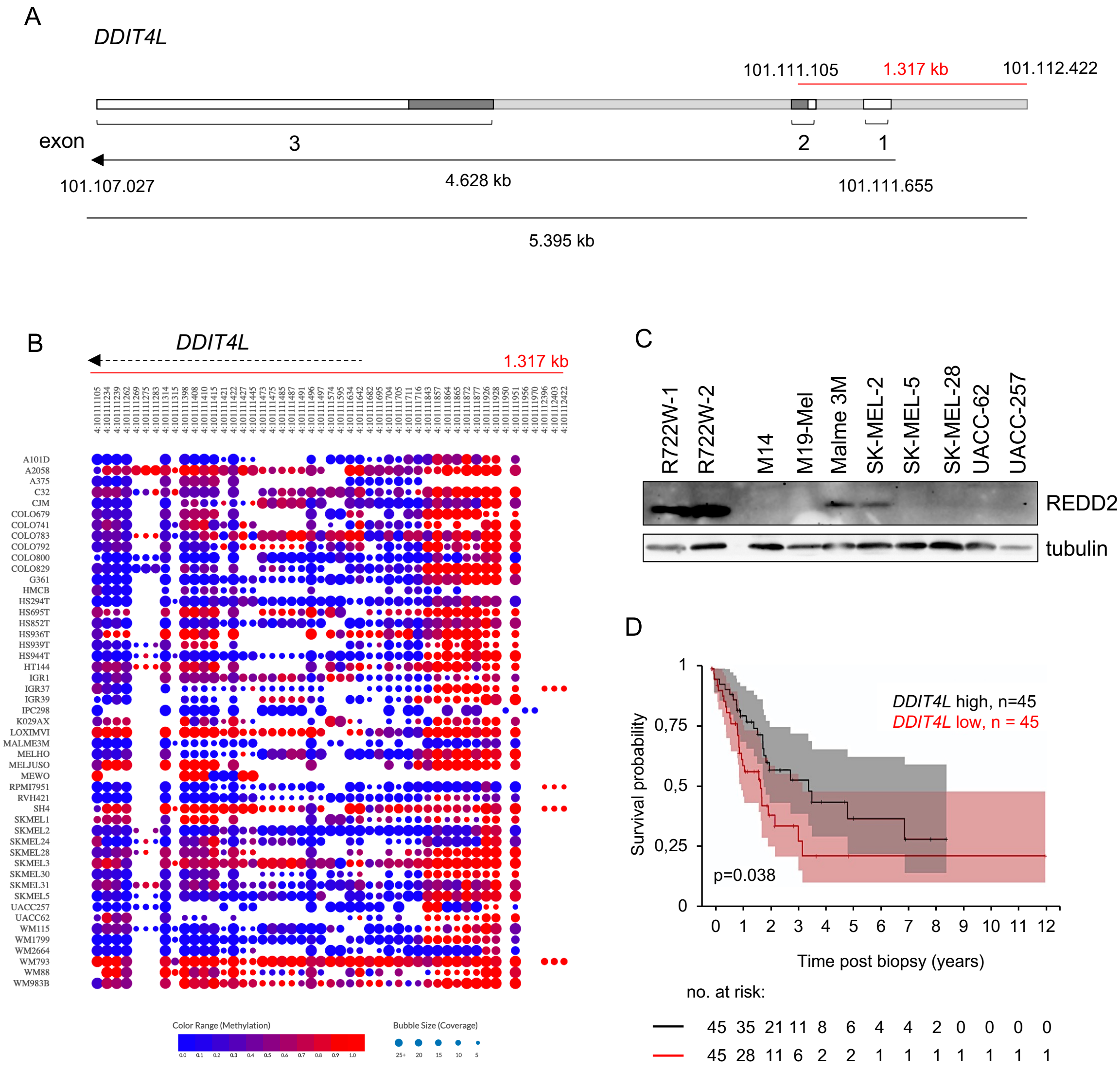

Supplementary Figure 3: DDIT4L in human melanoma

A: Schematic presentation of the human *DDIT4L* gene. Exons 1-3 are represented in dark grey (coding region) and white (non-coding region), introns are light grey. Numbers indicate the position of the respective gene region on chromosome 4 (based on GRCh37/hg19 genome assembly). The region marked in red is covered by the CpG analysis shown in **B**. **B:** CpG methylation in the *DDIT4L* promoter of 49 human melanoma cell lines. The analysis was conducted with https://depmap.org/portal/ (7). **C:** Western blot of DDIT4L in indicated melanoma cell lines. R722W WT31 cells were used as reference. Tubulin served as loading control. **D:** Overall survival probability of melanoma patients according to the expression of *DDIT4L* in the tumor (10% percentile), using the TCGA browser of the University of Zurich dataset (http://tcgabrowser.ethz.ch:3838/PROD/, TCGA dataset Skin Cutaneous Melanoma).

Supplementary Figure 4

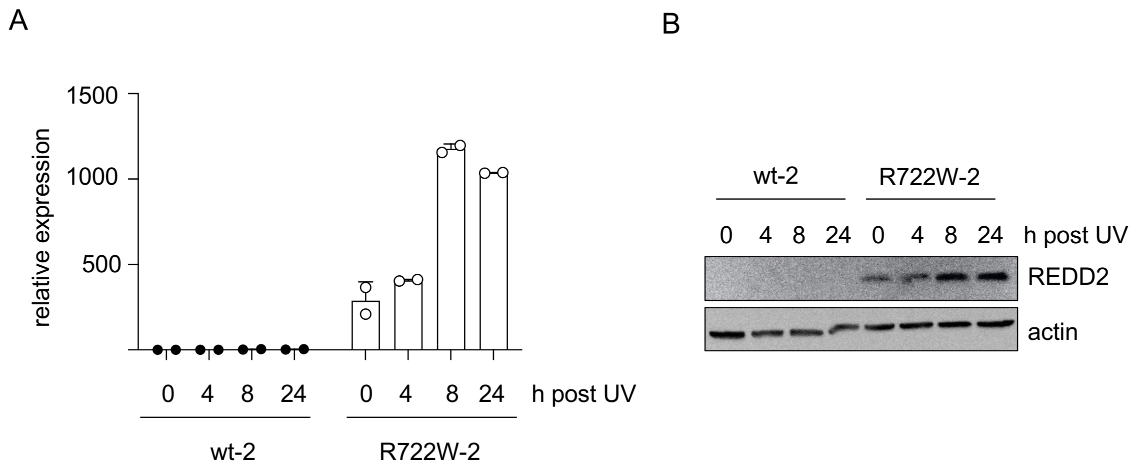

Supplementary Figure 4: UV-mediated REDD2 expression in TTD cells

A: Real-time PCR of *Ddit4l* in indicated murine WT31 melanoma cells after exposure to UV (1 mJ/cm^2). Data are derived from two independent samples. **B:** Corresponding western blot, using actin as loading control.

Supplementary Figure 5

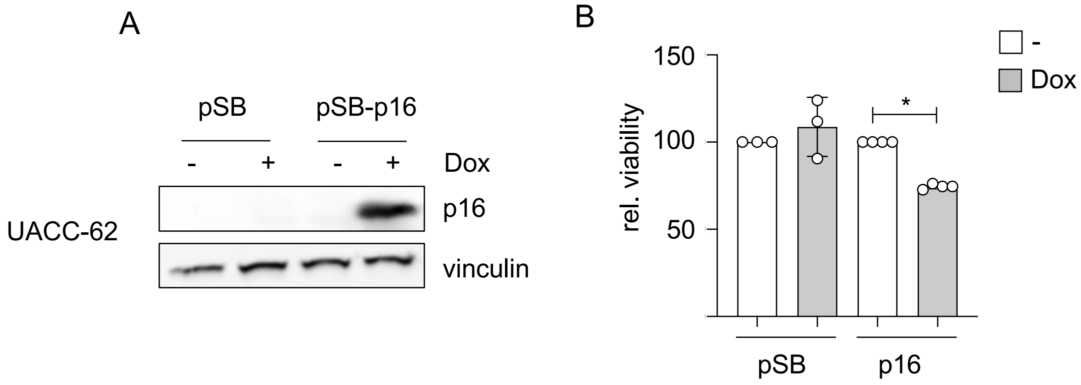

Supplementary Figure 5: Inhibitory effect of the common tumor suppressor p16 in human melanoma cells

A: Western blot of p16 protein expression in UACC-62 controls (expressing the empty vector control pSB) or p16-overexpressing cells (expressing pSB-p16) in response to doxycycline (100 ng/ml). **B:** Corresponding MTT viability assay, measured after 72 h. *: $p<0.05$ (Student`s t test, unpaired). Data are derived from 3-4 independent experiments, each done in triplicates.

Supplementary Figure 6

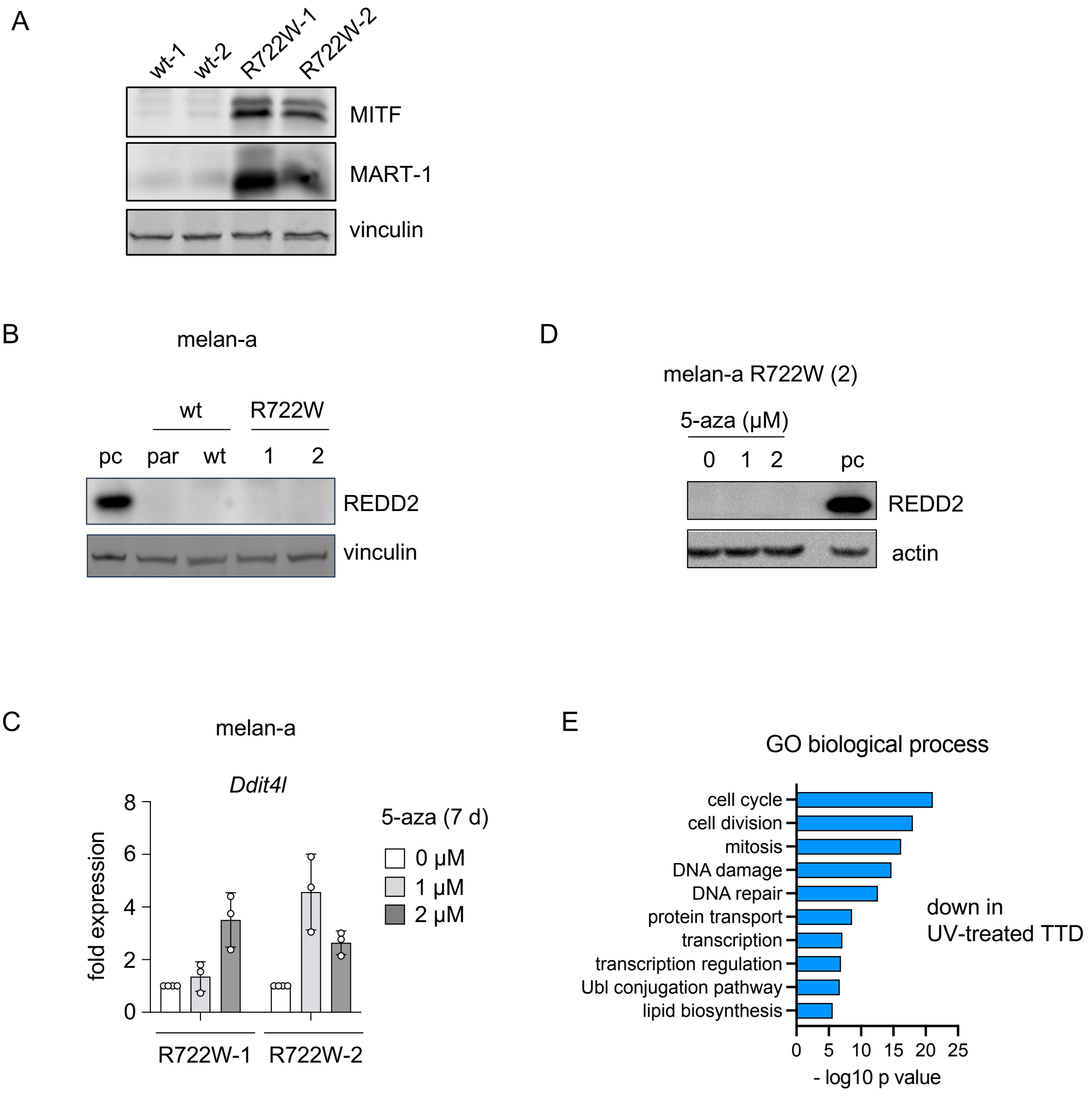

Supplementary Figure 6: Induction of MITF, but lack of REDD2 expression in melan-a TTD cells

A: Protein blot of MITF and MART-1 in two XPD-wt and two XPD-R722W clones (melan-a cell line). Vinculin served as loading control. **B:** Western blot of REDD2 in parental (par), XPD-wt (wt) or R722W melan-a cells under basal conditions. WT31 R722W cells served as positive control (pc). Vinculin was used as loading control. **C:** *Ddit4l* gene expression in melan-a R722W clones after 7 days of treatment with increasing concentrations of the DNA methylation inhibitor 5-azacytidine (5-aza). **D:** REDD2 western blot, showing lack of REDD2 expression in melan-a R722W cells after a 7-day treatment with 1 or 2 µM azacytidine, respectively. WT31

R722W cells served as positive control (pc). Actin was used as loading control. **E:** Top 10 significantly enriched gene groups specifically observed in UV-treated R722W melan-a cells (>2-fold change, $p<0.05$), but not UV-treated par or wt cells, according to the gene ontology category “biological process” (https://davidbioinformatics.nih.gov/). Please note that among the top 10, all categories entail downregulated genes.

Supplementary Figure 7

A

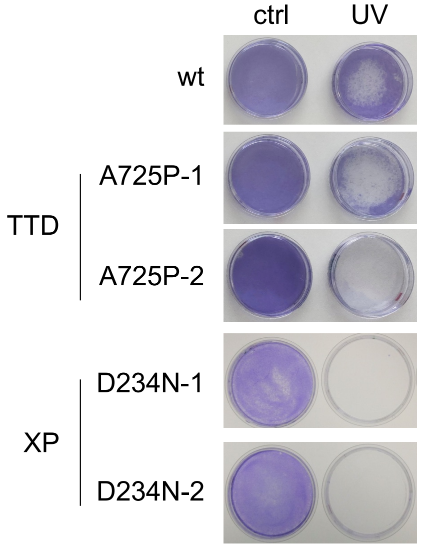

B

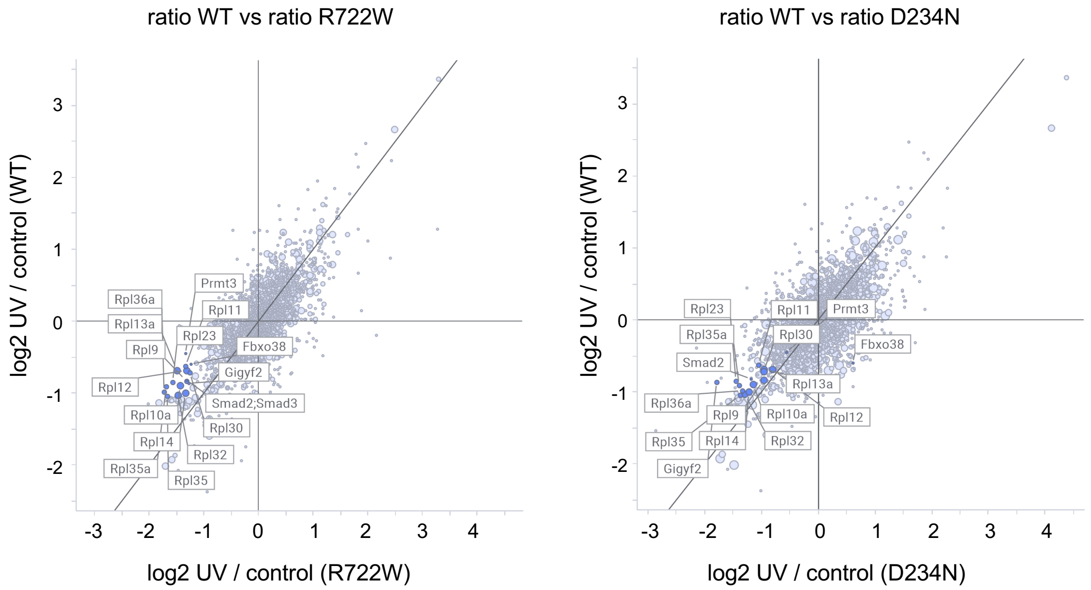

Supplementary Figure 7: Decrease of RPL proteins in WT31 melanoma cells after UV exposure

A: Crystal violet staining, showing the growth of indicated melan-a cells under control conditions or after exposure to a single dose of UV (1 mJ/cm^2), followed by an 8-day cultivation period. **B**: Scatter plot of the normalized log2 ratio of cellular proteins in UV-treated versus

control R722W melan-a cells (x axis) and XPD-wt cells (y axis) (left) or D234N melan-a cells and XPD-wt cells (right). The number of quantitative values (ratio count) per protein, used for quantification, is represented by the dot size. The lower left quadrant contains predominantly RPL proteins, which are downregulated particularly in R722W compared to XPD-wt cells.
