# Supplementary material for: Anti-tumorigenic properties by trichothiodystrophy mutations in melanocytic cells
Source: NAR Cancer. 2025 Aug 30;7(3):zcaf026. doi: 10.1093/narcan/zcaf026 (PMC12409403; doi:10.1093/narcan/zcaf026)
Supplement: zcaf026_Supplemental_Files [file zcaf026_supplemental_files.zip › Supplementary Table 1.docx]

**Supplementary Table 1**

Specifications of siRNAs used in this study.

| **target** | **company** | **name in manuscript** | **Order nr** |
| --- | --- | --- | --- |
| non-targeting | Sigma-Aldrich | siRNA ctrl | SIC001 |
| *Mitf* (mouse) | Sigma-Aldrich | si MITF #1 | SASI_Mm02_00307203 |
| *Mitf* (mouse) | Sigma-Aldrich | si MITF #2 | SASI_Mm02_00307204 |
| *Ddit4l* (mouse) | Sigma-Aldrich | si *Ddit4l* (pool of four) | SASI_Mm01_00163951 |
| *Ddit4l* (mouse) | Sigma-Aldrich | si *Ddit4l* (pool of four) | SASI_Mm01_00163952 |
| *Ddit4l* (mouse) | Sigma-Aldrich | si *Ddit4l* (pool of four) | SASI_Mm01_00163953 |
| *Ddit4l* (mouse) | Sigma-Aldrich | si *Ddit4l* (pool of four) | SASI_Mm01_00163954 |
