# Supplementary material for: Anti-tumorigenic properties by trichothiodystrophy mutations in melanocytic cells
Source: NAR Cancer. 2025 Aug 30;7(3):zcaf026. doi: 10.1093/narcan/zcaf026 (PMC12409403; doi:10.1093/narcan/zcaf026)
Supplement: zcaf026_Supplemental_Files [file zcaf026_supplemental_files.zip › Supplementary Table 2.docx]

**Supplementary Table 2**

**Oligonucleotides used in this study.**

1. Oligonucleotides for real-time PCR:

| **Gene** | **ENSEMBL ID** | **5`primer** | **3`primer** |
| --- | --- | --- | --- |
| *Actb*  *(mouse)* | ENSMUSG00000029580 | 5`-GCTACAGCTTCACCACCACA-3` | 5`- AAGGAAGGCTGGAAAAGAGC-3` |
| *Ddit4l*  *(mouse)* | ENSMUSG00000046818 | 5`-CACCCTGGGAGTCTGCTAAG-3’ | 5`-GGTCAGTTTCTCAGGGACCA-3` |
| *Mreg*  *(mouse)* | ENSMUSG00000039395 | 5`-ATGACGTGTCCCACACAGAG-3` | 5`-TCTCCATCGGTTCCTCACTT-3` |
| *Myo5a*  *(mouse)* | ENSMUSG00000034593 | 5-  TTTGGATCCCTGATCCTGAG-3` | 5`-  TCAGGGTTCCGTAAGTGAGG-3` |
| *Tyrp1*  *(mouse)* | ENSMUSG00000005994 | 5`-  TCTGGCCTCCAGTTACCAAC-3` | 5`-  TCAGTGAGGAGAGGCTGGTT-3` |
| *Rab38*  *(mouse)* | ENSMUSG00000030559 | 5`-  GTGGGCAAGACCAGCATTAT-3` | 5`-  CCCATAGCTTCCCGGTAATA-3` |

1. Oligonucleotides for cloning:

| **Primer ID** | **Purpose** | **Sequence** |
| --- | --- | --- |
| hERCC2_NheI_5` | Cloning of human ERCC2 into pSB | 5`- GCGGCTAGCATGAAGCTCAACGTGGACGGG -3` |
| hERCC2_AfeI_3` | “ | 5`- GCGAGCGCTTCAGAGCTGCTGAGCAATCTG -3 |
| mDdit4l_AfeI_5` | Cloning of murine Ddit4l into pSB | 5`-GCGAGCGCTATGGTTGCAACGGGCAGTTTG-3` |
| mDdit4l_BstBI_3` | “ | 5’-GCGTTCGAATTAGCACTCTTCAATGACTGTC -3’ |
| h_ERCC2_R722W_5' | Site-directed mutagenesis (R722W) | 5'-GTGGCCAAGTACTTCCTGTGGCAGATGGCACAGCCCT -3’ |
| h_ERCC2_R722W_3' | “ | 5'-AGGGCTGTGCCATCTGCCACAGGAAGTACTTGGCCAC-3` |
| h_ERCC2_D234N_5' | Site-directed mutagenesis (D234N) | 5’-CGTGGTCTTCAACGAGGCCCACAACATTG-3’ |
| h_ERCC2_D234N_3' | “ | 5’- ACGGCCTTGCGGGCCAGT-3’ |
| h_ERCC2_A725P_5` | Site-directed mutagenesis (A725P) | 5`-GCGGCAGATGcCACAGCCCTTCCA-3` |
| h_ERCC2_A725P_5` | “ | 5`-AGGAAGTACTTGGCCACCTG-3` |
| gRNA_Ercc2_#1_5` | Cloning of gRNA vector for CRISPR/Cas9 knockout (murine Ercc2) | 5`CACCGGAACCTGTGCATTCATCCCG -3` |
| gRNA_Ercc2_#1_2` | “ | 5`-AAACCGGGATGAATGCACAGGTTCC-3` |
| gRNA_Ercc2_#2_5` | “ | 5`-CACCGCCCACAGGCTTATCCGCTGG-3` |
| gRNA_Ercc2_#2_3` | “ | 5`-AAACCCAGCGGATAAGCCTGTGGGC-3` |
